# Autophagy-associated circRNA circCDYL augments autophagy and promotes breast cancer progression

**DOI:** 10.1186/s12943-020-01152-2

**Published:** 2020-03-25

**Authors:** Gehao Liang, Yun Ling, Maryam Mehrpour, Phei Er Saw, Zihao Liu, Weige Tan, Zhenluan Tian, Wenjing Zhong, Wanyi Lin, Qing Luo, Qun Lin, Qiufang Li, You Zhou, Ahmed Hamai, Patrice Codogno, Jun Li, Erwei Song, Chang Gong

**Affiliations:** 1 Breast Tumor Center, Guangdong Provincial Key Laboratory of Malignant Tumor Epigenetics and Gene Regulation, Sun Yat-sen Memorial Hospital, Sun Yat-sen University, 107 Yanjiang West Road, Guangzhou, 510120 China; 2 Institut Necker-Enfants Malades (INEM), Inserm U1151-CNRS UMR 8253, 75993 Paris, France; 3 Université Paris Descartes-Sorbonne Paris Cité, 75993 Paris, France; 4 Medical Research Center, Guangdong Provincial Key Laboratory of Malignant Tumor Epigenetics and Gene Regulation, Sun Yat-sen Memorial Hospital, Sun Yat-sen University, Guangzhou, 510120 China; 5 Department of Breast Surgery, the First Affiliated Hospital of Guangzhou Medical University, Guangzhou Medical University, Guangzhou, 510120 China; 6 Systems Immunity University Research Institute and Division of Infection and Immunity, School of Medicine, Cardiff University, Cardiff, CF14 4XN UK; 7 Minerva Foundation Institute for Medical Research, 00290 Helsinki, Finland; 8 Department of Biochemistry, Zhongshan School of Medicine, Sun Yat-sen University, Guangzhou, 510080 China; 9 Program of Molecular Medicine, Zhongshan School of Medicine, Sun Yat-Sen University, Guangzhou, 510080 China; 10 Guangzhou Regenerative Medicine and Health Guangdong Laboratory, Guangzhou, 510005 China

**Keywords:** circCDYL, Autophagy, Breast cancer, miRNA sponge

## Abstract

**Background:**

Although both circular RNAs (circRNAs) and autophagy are associated with the function of breast cancer (BC), whether circRNAs regulate BC progression via autophagy remains unknown. In this study, we aim to explore the regulatory mechanisms and the clinical significance of autophagy-associated circRNAs in BC.

**Methods:**

Autophagy associated circRNAs were screened by circRNAs deep sequencing and validated by qRT-PCR in BC tissues with high- and low- autophagic level. The biological function of autophagy associated circRNAs were assessed by plate colony formation, cell viability, transwells, flow cytometry and orthotopic animal models. For mechanistic study, RNA immunoprecipitation, circRNAs pull-down, Dual luciferase report assay, Western Blot, Immunofluorescence and Immunohistochemical staining were performed.

**Results:**

An autophagy associated circRNA circCDYL was elevated by 3.2 folds in BC tissues as compared with the adjacent non-cancerous tissues, and circCDYL promoted autophagic level in BC cells via the miR-1275-ATG7/ULK1 axis; Moreover, circCDYL enhanced the malignant progression of BC cells in vitro and in vivo. Clinically, increased circCDYL in the tumor tissues and serum of BC patients was associated with higher tumor burden, shorter survival and poorer clinical response to therapy.

**Conclusions:**

circCDYL promotes BC progression via the miR-1275-ATG7/ULK1-autophagic axis and circCDYL could act as a potential prognostic and predictive molecule for breast cancer patients.

## Background

Breast cancer (BC) is the most common malignancy in women worldwide [1]. Though BC patients have favorable prognosis due to recent advances in early diagnosis and effective treatment, recurrence or metastasis remained to threaten survival. Autophagy is a conserved ubiquitous process and energy recycling system that delivers damaged organelles, misfolded proteins and intracellular constituents to lysosomes for degradation [2]. Dysregulation of autophagy causes various pathological behaviors in eukaryotic cells and promotes progression of diseases [3], including cancer [4–6]. Autophagy provides energy for cancer cells in stress condition (i.e. hypoxia, starvation) and helps sustain their survival [7]. It has been reported that autophagy promotes survival, proliferation, metastasis, and invasion of BC cells via regulating autophagy associated genes and non-coding RNAs [4, 8–12]. However, the underlying mechanisms, especially non-coding RNAs, in regulating autophagy of BC progression are not yet fully elucidated.

Circular RNAs (circRNAs) are a class of novel RNAs with covalently closed loop structure without 5′ caps and 3′ tails [13]. Due to its loop structure, circRNAs are resistant to exonuclease, so they are much more stable than their parental linear RNAs and are abundant in mammalian cells [14]. circRNAs display disease-specific and development stage-specific characteristics under different pathologic environments [15–17], which suggests that circRNAs can be used as potential biomarkers for diagnosis and therapy [18, 19]. Several studies demonstrated that circRNAs could act as miRNA sponges [7, 20–23], and circRNAs with intronic sequence could regulate transcription of parental genes by interacting with RNA polymerase II in the nucleus [24, 25], while certain circRNAs exhibited the ability to translate proteins [26–28].

The most frequently reported mechanism of circRNAs is to trap miRNAs, known as miRNA sponge, leading to functional loss of target miRNAs and subsequent upregulation of miRNA-targeted genes. circRNAs exhibit powerful potential in regulating the biological functions of BC cells, including proliferation, migration, invasion [29–31]. Recently, some publications reported correlations between circRNAs and autophagy in many diseases such as thyroid cancer, myocardial ischemia/reperfusion injury and sciatic nerve injury [10, 32, 33]. For example, circ-Dnmt1 promoted the proliferation of BC cells via autophagy by interfering with the nuclear localization of p53 and AUF1 [10]. Nevertheless, the relationship between circRNAs and autophagy in breast cancer, as well as the role of autophagy-associated circRNAs in clinical diagnosis and treatment of breast cancer remain largely illusive.

In this study, by using deep sequencing in BC tissues, we identified an autophagy associated circRNA circCDYL, which was increased up to 3.2 folds in BC tissues than that of adjacent non-cancerous tissues. Although circCDYL is reported to be highly expressed in hepatocellular carcinoma [34], bladder cancer [35] and breast cancer [36], the functional role of circCDYL in breast cancer still remains unknown. Herein, we found that BC patients with higher circCDYL in the serum or tumor tissues had shorter survival and poorer clinical response to therapy. We further revealed that circCDYL promoted autophagy level via sponging miR-1275 and up-regulated the expression of autophagy-associated gene ATG7 and ULK1. We demonstrated that circCDYL promoted BC progression via autophagy both in vitro and in vivo.

## Material and methods

### Patient samples and clinical database

In this study, three independent cohorts of BC patients from Sun Yat-sen Memorial Hospital (SYSMH) were enrolled. Breast cancer patients (aged 32 to 81) without any distant metastasis at first diagnosis between 1 June 2004 and 31 May 2018 were enrolled as Cohort 1 (*n* = 113). Paraffin-embedded BC tissue samples and paired non-tumor tissue samples in Cohort 1 were collected for in situ hybridization (ISH), immunohistochemical staining (IHC) or immunofluorescence (IF). In addition, serum samples from two other cohorts of SYSMH were collected for droplet digital PCR (ddPCR) detection (Cohort 2 was a retrospective cohort collected from 1 March 2017 to 30 September 2017, containing 14 benign patients, 30 early breast cancer (EBC) patients and 18 metastatic breast cancer (MBC) patients; Cohort 3 was a prospective cohort from 1 June 2015 to 1 April 2019, in which only MBC patients at first diagnosis were enrolled). This work was approved by Sun Yat-sen Memorial Hospital Ethics Committee (SYSY-KY-KS-2018-05).

### Autophagosome detection in cell lines and paraffin-embedded section of tissues

LC3 (MAP 1LC3B, Microtubule Associated Protein 1 Light Chain 3 Beta) is a recognized autophagic marker. During the formation of autophagosomes, cytosolic LC3 (LC3-I) would be enzymatically cleaved and form membrane-bound LC3 (LC3-II), which mainly locates in autophagosomes. Therefore LC3-II is a hallmark for autophagosome formation. In our study, mCherry-GFP-LC3-labeled MDA-MB-231 was used to visualize autophagosome and autolysosome. Since GFP is quenched in autolysosome, green dots of unmerged pictures reflect only autophagosomes, while red dots pictures reflect both autophagosomes and autolysosomes. Therefore, yellow and red dots of the merged picture reflect autophagosomes and autolysosomes, respectively. For the immunofluorescence on paraffin-embedded sections of human or animal tissue, the LC3 antibody were used to detect the autophagosomes, which could be seen as LC3 dots. Autophagosomes of cell lines or tissue sections were observed under confocal microscope in five random fields and the average number of autophagic dots in each cell were calculated to represent its autophagic level.

### circRNA pull-down

circRNA pull-down was performed using biotinylated circCDYL probe, linear CDYL probe and negative control (NC) probe (Sangon Biotech, China) based on protocols from previous literatures [32, 37]. Briefly, MDA-MB-231 was fixed with 1% formaldehyde for 30 mins and lysed by co-IP buffer. The mixture was sonicated at high amplitude for 30 cycles of 30 s (on/off) pulses. Then the circCDYL-specific and NC biotinylated probes were added to the supernatant and the mixtures were incubated overnight at 37 °C. Next, the mixture was incubated with C1 streptavidin magnetic beads for 30 min at 37 °C. Finally, total RNA in the solution was extracted followed by qRT-PCR detection of circCDYL, linear CDYL RNA and miR-1275.

### Dual luciferase reporter assay

Full-length sequence with wild-type or mutant sites of circCDYL and linear CDYL were designed and inserted into psiCHECK-2 vectors (Synbio-tech, Guangzhou, China). psiCHECK-2 vectors carrying NC mimic or miR-1275 mimic were co-transfected to HEK-293 T cells respectively. After 48 h transfection, luciferase activity of the transfected cells was detected by the dual-luciferase reporter assay system (Vazyme, Nanjing, China).

### In vivo breast cancer orthotopic model

The animal experiments were performed by Forevergen Medical Corporation (Guangzhou, China), and all experimental procedures and animal care were in accordance with the ethical guidelines of the institution. 4-week-old female Balb/c nude mice were purchased from Nanjing Biomedical Research Institute of Nanjing University (Nanjing, China). Luciferase-labeled MDA-MB-231 cells (1X 10^6^) transduced with sh-NC or sh-circCDYL lentivirus (sh-circCDYL-1 or sh-circCDYL-2) were directly injected into the fourth left mammary fat pads of the nude mice (*n* = 6/group). The tumor size was measured every 3 days by using Vernier caliper. When the tumor volume of sh-NC group reached 1000 mm^3^, mice were given D-luciferin (300 mg/kg; *i.p.;* 10 mins prior to imaging), anaesthetized with 3% isoflurane and imaged with a Xenogen IVIS Lumina system (Caliper Life Sciences, Hopkinton, MA). The mice were then sacrificed and the tumors were resected for IHC staining, ISH and IF.

### Statistical analysis

Statistical analyses were performed using GraphPad Prism 5 software. Student’s *t*-test or one-way ANOVA was performed to test differences between groups in both in vivo and in vitro experiments. *X*^*2*^-test was applied to analyze the correlations between circCDYL expression and clinicopathological characterization of BC patients. Kaplan-Meier plots and Log-rank tests were used for survival analysis. The univariate analyses were analyzed by Cox proportional hazards model. The correlations were analyzed using Pearson’s correlation coefficients. *P* <  0.05 was considered statistically significant.

### Additional material and methods

Additional materials and methods can be found in Supplementary Information.

## Results

### Identification of autophagy-associated circRNAs in breast cancer

**Fig. 1:**
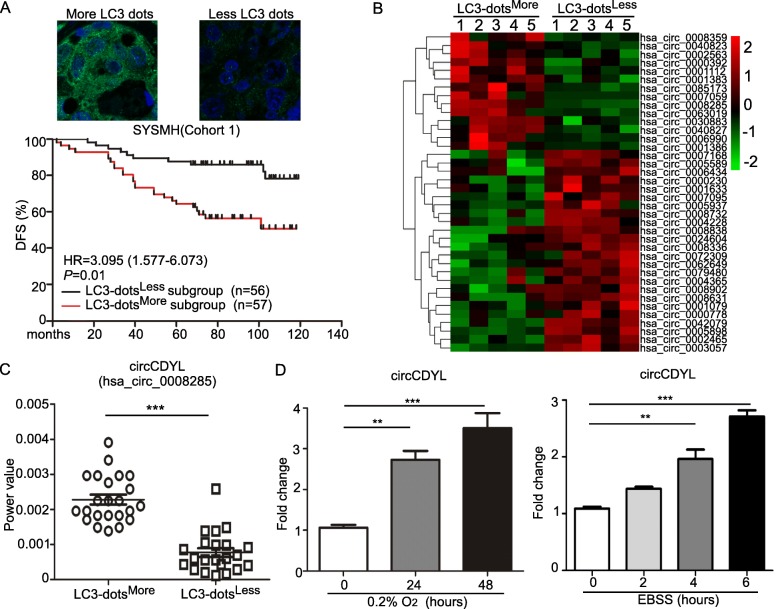
Autophagy-associated circRNA (circCDYL) expression profile in breast cancer. **a** The association between LC3 dots and disease-free survival (DFS) in 113 breast cancer patients from Cohort 1 of SYSMH. **b** circRNA expression profile in breast cancer patients with more and less LC3 dots by circRNA deep sequencing. **c** Expression of circCDYL in breast cancer tissue with more (*n* = 23) and less (*n* = 22) LC3 dots by qRT-PCR. **d** The relative expression of circCDYL under hypoxic condition (0.2% O_2_) at various time points (0, 24, 48 h) (left) and EBSS starvation induction at various time points (0, 2, 4, 6 h) (right) in MDA-MB-231 cells

In order to determine the clinical significance of autophagy in breast cancer, the expression of LC3 detected by IHC in breast cancer patients was analyzed in Cohort 1. The median value of LC3 level was used as a cut-off value to divide these patients into LC3^High^ and LC3^Low^ subgroups. Survival analysis revealed that patients in LC3^High^ subgroup had a poorer disease-free survival (DFS) than LC3^Low^ subgroup (HR 2.069, 95% CI 1.059–4.044, *P* = 0.033, Fig. S1A). However, IHC staining on tumor section only reflected the total protein expression level of LC3, but not LC3-II (An acknowledged hallmark of autophagosome). Therefore, detecting LC3 dots by immunofluorescence (IF) was visible and allowed us to calculate autophagosomes. The median number of LC3 dots was used as a cut-off value to divide Cohort 1 into LC3-dots^More^ and LC3-dots^Less^ subgroups. Survival analysis showed that LC3-dots^More^ subgroup had a poorer DFS (HR 3.095, 95% CI 1.577–6.073, *P* = 0.01, Fig. 1a). These results indicated that autophagy plays an important role in breast cancer progression.

To further screen autophagy-associated circRNAs in breast cancer, circRNAs deep sequencing was performed to compare the difference of circRNAs profiles in primary BC tissues with more (LC3-dots^More^, *n* = 5) and less (LC3-dots^Less^, n = 5) LC3 dots (Fig. 1b). 38 circRNAs candidates were selected based on the following standards: (i) fold change > 2; (ii) Junction reads per million mapped reads (RPM) > 200 (Table S1). From these candidates, we further narrow down the selection by selecting top ten circRNAs. Then we performed qRT-PCR to further quantify this result in LC3-dots^More^ (*n* = 23) and LC3-dots^Less^ BC tissues (*n* = 22), and found that only three circRNA candidates - hsa_circ_0008285 (derived from CDYL gene, hereafter termed as circCDYL), hsa_circ_0024604 and hsa_circ_0007059 were consistent with the result of circRNAs deep sequencing (Fig. 1c, Fig. S1B). Next, we established two autophagy induction breast cancer cell models in vitro under hypoxia and EBSS starvation treatment (Fig. S2A-2C), and found that only circCDYL was up-regulated in both models (Fig. 1d, Fig. S2D-E). All these data above indicated that circCDYL is an autophagy associated circRNA, and the basic characteristic of circCDYL has been reported in published research [34].

### High circCDYL is associated with poor prognosis and clinical response in BC patients

**Fig. 2:**
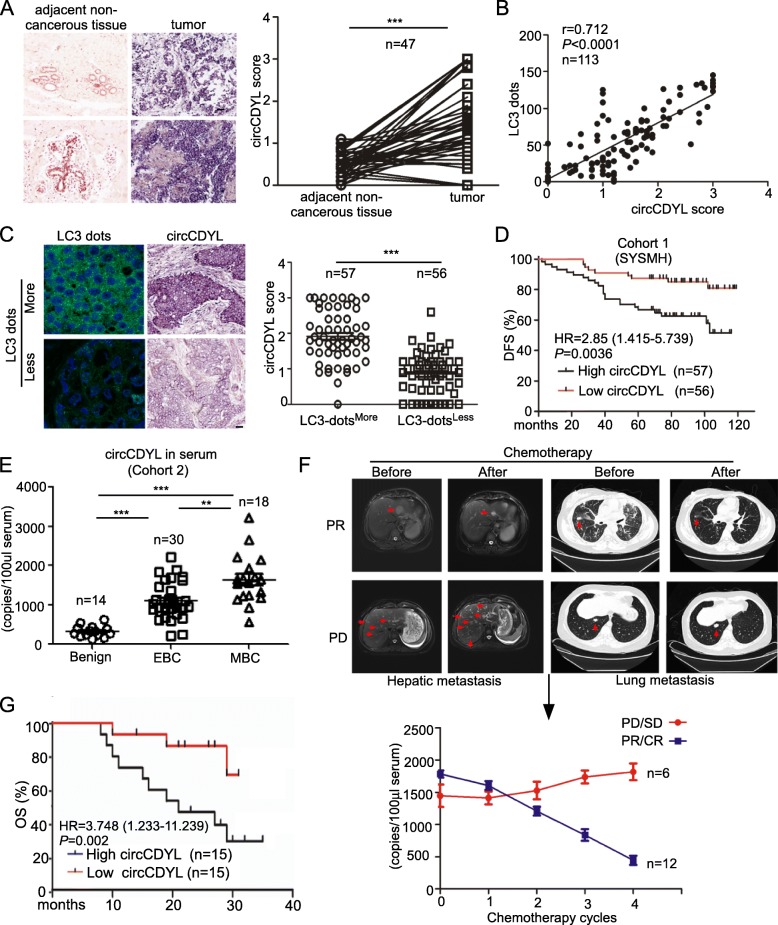
Clinical significance of circCDYL in breast cancer. **a** Comparison of circCDYL expression between breast cancer tissues and paired adjacent non-cancerous tissues by qRT-PCR (*n* = 47) and their representative images by ISH. Scale bar = 50 μm. **b** Correlations of circCDYL expression and the number of LC3 dots in breast cancer tumors, analyzed by Pearson analysis. **c** Representative images of LC3 dots and circCDYL expression by ISH in breast cancer tissues (left) and quantitative analysis of circCDYL expression in cancer tissues with more and less LC3 dots. Scale bar = 50 μm. **d** Kaplan-Meier analysis of the correlation between circCDYL expression and disease-free survival (DFS). **e** circCDYL abundance in serum of patients detected by ddPCR (*n* = 62, Cohort 2). *EBC: early breast cancer, MBC: metastatic breast cancer.***f** Representative images in cases with lung and liver metastasis before and after chemotherapy and real time kinetics of serum circCDYL during chemotherapy in MBC patients (*n* = 18, Cohort 2). *PR: partial response; CR: complete response; PD: progressive disease; SD: stable disease*. **g** Kaplan-Meier analysis of the correlation between serum circCDYL expression and overall survival (OS) in MBC patients (Cohort 3). *P <  0.05, **P < 0.01, ***P < 0.005. Error bars indicate Standard Error of Mean (S.E.M)

The expression of circCDYL and linear CDYL in 113 BC tissues and 47 corresponding paired non-cancerous tissues from Cohort 1 were detected by ISH. circCDYL and linear CDYL was elevated up to 3.2 and 1.5 folds respectively in BC tissues than adjacent non-cancerous tissues (Fig. 2a, Fig. S3A). The clinicopathological features were analyzed based on expression of circCDYL or linear CDYL in BC tissues (Table 1, Table S2). Higher circCDYL expression was significantly associated with ER negative status, higher Ki67 index, larger tumor size and more lymphatic metastasis. No correction was found between linear CDYL and these clinicopathological features. In addition, the Pearson correlation analysis showed that circCDYL and linear CDYL expression was positively correlated with the number of LC3 dots (circCDYL: r = 0.712, linear CDYL: r = 0.298) (Fig. 2b, Fig. S3B). The expression of circCDYL and linear CDYL was increased up to 2.2 and 1.2 folds in LC3-dots^More^ tissues, when comparing with LC3-dots^Less^ tissues (Fig. 2c, Fig. S3C**)**. A Kaplan-Meier analysis indicated that patients with high circCDYL had a poorer disease-free survival (DFS) (HR 2.85, 95% CI 1.415–5.739, *P* = 0.0036, Fig. 2d), but expression of linear CDYL had no effect on DFS of BC patients (Fig. S3D). These results suggested that circCDYL may play more important role in breast cancer than linear CDYL.

**Table 1:** Patient characteristics stratified by circCDYL expression

	Number of patients		Total	*P* value
	Low circCDYL	High circCDYL		
**Age**				0.728
>35	50	52	102	
≤ 35	6	5	11	
**Menopause**				0.644
Yes	35	38	73	
No	21	19	40	
**ER status**				0.035
Negative	14	25	39	
Positive	42	32	74	
**PR status**				0.106
Negative	30	39	69	
Positive	26	18	44	
**HER2 status**				0.20
Negative	38	32	70	
Positive	18	25	43	
**Ki67 Level**				< 0.0001
Low	48	20	68	
High	8	37	45	
**Molecular subtype**				0.102
Luminal	42	32	74	
HER2+	7	11	18	
TNBC	7	14	21	
**Tumor stage**				0.001
T1	27	11	38	
T2	23	28	51	
T3–4	6	18	24	
**Lymphatic stage**				0.046
N0	28	17	45	
N1	16	17	33	
N2–3	12	23	35	

*Abbreviations: ER* estrogen receptor, *PR* progesterone receptor, *HER2* human epidermal growth receptor 2

Next, BC patients’ serum from Cohort 2 and Cohort 3 were collected. Droplet digital PCR (ddPCR) were performed to verify the abundance of circCDYL in serum of these patients. Clinical data from Cohort 2 showed that circCDYL was the most abundant in the serum from metastatic breast cancer (MBC) patients, compared to early breast cancer (EBC) and benign patients (Fig. 2e). We further detected the real time kinetics of serum circCDYL in MBC patients (*n* = 18) during chemotherapy and found that reduction of circCDYL is positively correlated to a better sensitivity to clinical therapy (Fig. 2f). In addition, survival analysis from Cohort 3 containing 30 MBC patients showed that patients with high serum circCDYL had a poorer overall survival (HR 3.748, 95% CI 1.233–11.239, *P* = 0.002, Fig. 2g). These clinical data suggested that circCDYL may be a potential molecule for predicting the prognosis and therapy response of BC patients.

### circCDYL regulates the proliferation of BC cells via autophagy

**Fig. 3:**
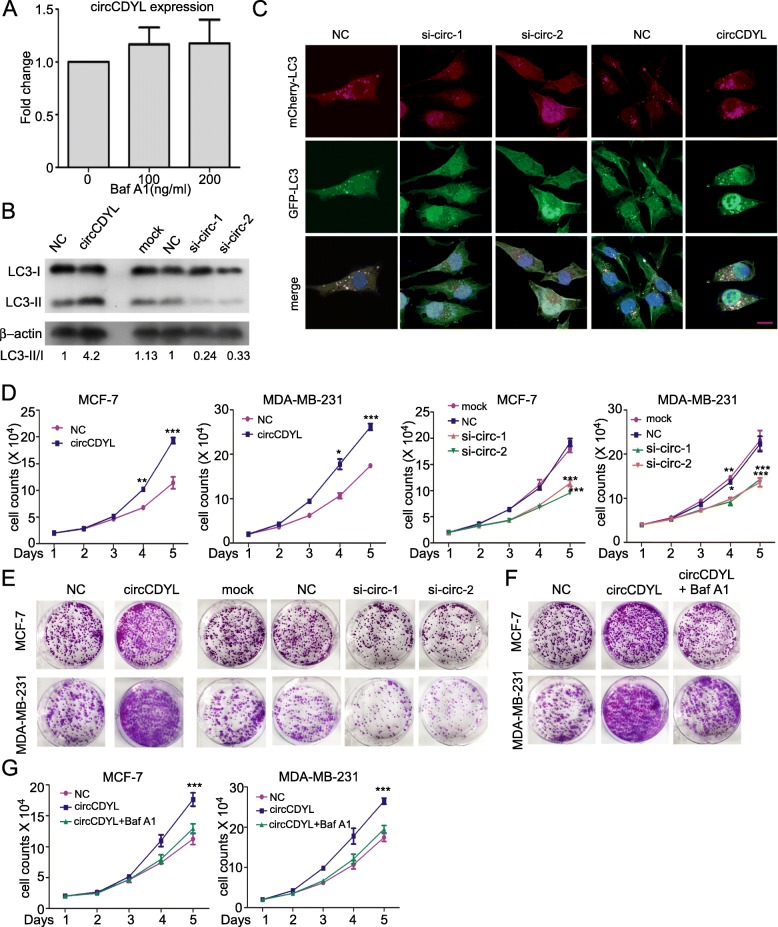
circCDYL promotes proliferation of breast cancer cells via autophagy. **a** qRT-PCR analysis of circCDYL in MDA-MB-231 cells after Baf A1 (Bafilomycin A1) treatment. **b** LC3 expression in MDA-MB-231 after circCDYL silencing via siRNA or over-expressing circCDYL by over-expressing plasmid, as detected by Western Blot. LC3-II/I, the gray value ratio of LC3-II and LC3-I, normalized to NC group. **c** Autophagosomes in mCherry-GFP-LC3 labeled MDA-MB-231 after silencing circCDYL via siRNA or over-expressing circCDYL by over-expressing plasmid, as detected by confocal microscopy. Scale bar = 50 μm. **d**, **e** The proliferation of MDA-MB-231 and MCF-7 cells after silencing circCDYL viasiRNA or over-expressing circCDYL via over-expressing plasmid, as detected by cell viability assay (**d**) and plate colony formation (**e**). **f**, **g** Proliferation of MDA-MB-231 and MCF-7, as detected by plate colony formation (**f**) and cell viability assay (**g**) after transfection with circCDYL over-expressing plasmid or co-treatment with Baf A1. siNC: negative control siRNA. All experiments above were repeated at least 5 times. *P < 0.05, **P < 0.01, ***P < 0.005. Error bars indicate S.E.M

To investigate the biological function of circCDYL, the expression of circCDYL was downregulated by RNAi-mediated gene silencing using siRNA (Fig. S4A) and lenti-shRNA (Fig. S4B). Overexpression of circCDYL was carried out through transfection of circCDYL over-expressing plasmids (Fig. S4C) or circCDYL over-expressing lentivirus (Fig. S4D) without influencing the expression of linear CDYL RNA. Next, we found that inhibition of autophagy by bafilomycin A1 (Baf A1, an autophagy inhibitor) had no influence on the expression level of circCDYL in MDA-MB-231 cells (Fig. 3a, Fig. S4E). However, silencing or over-expression of circCDYL resulted in down- or up- regulation of the basal autophagic level of MDA-MB-231 cells as determined by LC3-II protein level (Fig. 3b). To visualize autophagosomes, we then established a stable MDA-MB-231 cell line with LC3 labeled by mCherry and GFP. LC3 dots were increased after circCDYL overexpression in LC3-mCherry-GFP-labeled MDA-MB-231 cells, and decreased after circCDYL silencing (Fig. 3c, Fig. S4F). All these indicate that circCDYL affects autophagic level in BC cells, but autophagy did not affect the expression of circCDYL.

Next, we investigated the biological function of circCDYL in BC cells. Cell viability assays indicated that the proportion of living cells were increased after circCDYL overexpression, and significantly decreased after siRNA silencing of circCDYL in both MCF-7 and MDA-MB-231 (Fig. 3d). Similarly, plate colony formation assay showed that colony formation increased after circCDYL overexpression and decreased after circCDYL silencing (Fig. 3e, Fig. S4G). However, circCDYL expression level had no significant effect on the invasion and apoptosis in MDA-MB-231 cells (Fig. S4H-I). Both plate colony formation assay and cell viability assay showed that inhibition of autophagic flux by Bafilomycin A1 attenuated the effect of circCDYL overexpression on proliferation in MDA-MB-231 and MCF-7 cell lines (Fig. 3f-g, Fig. S4J).

In addition, we designed three siRNAs: (i) target against circCDYL only (si-circ), (ii) target against linear CDYL only (si-linear), and (iii) target against both circCDYL and linear CDYL (si-cl) (Fig. S5A). Both si-circ and si-cl transfection had similar effect on the decreased proliferation of MDA-MB-231 and MCF-7 cells, while si-linear transfection had no significant effect on cell proliferation (Fig. S5B). In addition, both si-circular and si-cl transfection increase autophagic level by detecting protein level of LC3-II, while si-linear showed little effect on autophagy regulation (Fig. S5C). The result above indicates that circCDYL but not linear CDYL has the biological function on proliferation and autophagy regulation.

### circCDYL works as miR-1275 sponge in breast cancer cells

**Fig. 4:**
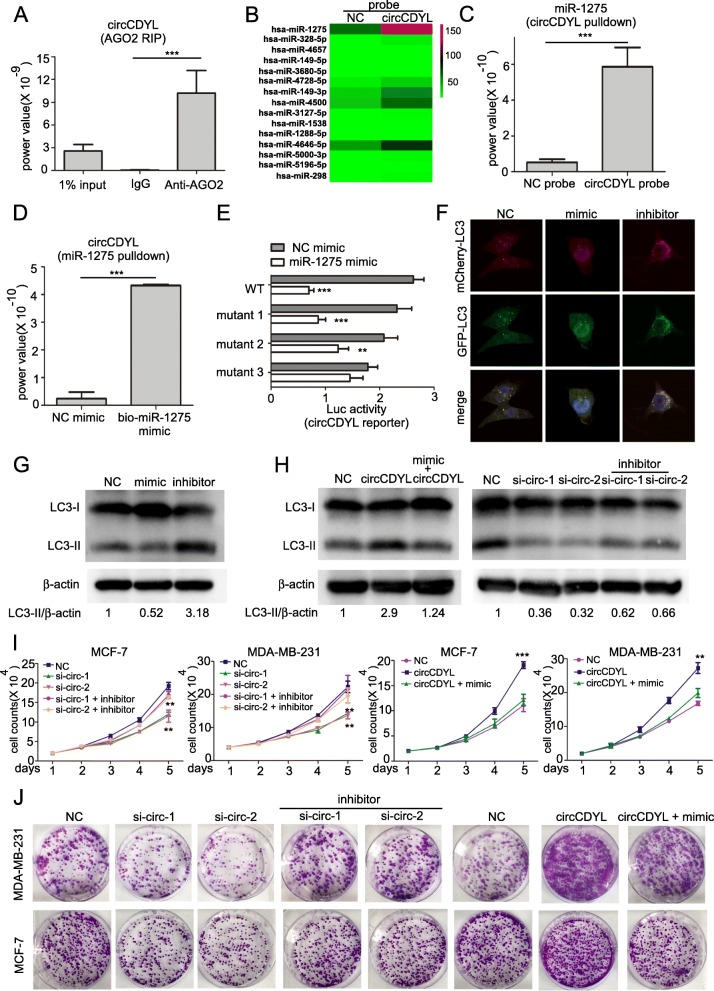
circCDYL functions as miR-1275 sponge. **a** qRT-PCR analysis of circCDYL in RNA sample after RIP assay by AGO2 antibody. **b** Heatmap of miRNA microarray of RNA sample by circCDYL pull-down. **c** qRT-PCR analysis of miR-1275 in RNA sample by circCDYL pull-down. **d** qRT-PCR analysis of circCDYL in RNA sample by miR-1275 miRNA pull-down. **e** Dual luciferase assay of HEK-293 T cells co-transfected with miR-1275 mimic and luciferase reporter containing full length of circCDYL with or without miR-1275 binding site mutant. *NC: negative control; WT: wild-type luciferase reporter containing full length of circCDYL*. **f** LC3 dots in mCherry-GFP-LC3-labeled MDA-MB-231 cells. **g** LC3-II expression in MDA-MB-231 after treatments with miR-1275 mimic or inhibitor, as detected by Western Blot. **h** Western Blot analysis of LC3-II expression in MDA-231 co-transfected with circCDYL over-expressing plasmid and miR-1275 mimic or co-transfected with circCDYL specific siRNA and miR-1275 inhibitor. **i-j.** Proliferation level of MCF-7 and MDA-MB-231 detected by cell viability assay (**i**) and plate colony formation (**j**) after co-transfection with circCDYL over-expressing plasmid and miR-1275 mimic or co-transfection with circCDYL specific siRNA and miR-1275 inhibitor. All data are shown as the mean ± S.E.M. *P < 0.05, **P < 0.01, ***P < 0.005

It has been demonstrated that circRNAs have a potential to work as a miRNA sponge if the circRNAs can form a circRNA-AGO2-miRNA complex by binding to AGO2 and miRNAs [22, 23, 38]. AGO2 RIP assay confirmed that AGO2 could bind circCDYL (power value =10.7*10^− 9^, Fig. 4a), suggesting that circCDYL may work as a miRNA sponge. We successfully established circRNAs pull-down assay using biotinylated probes specifically against circCDYL (Fig. S6A). We purified the circCDYL-binding RNAs by circRNAs pull-down and scanned the candidate miRNAs by miRNAs microarray (Fig. 4b, Table S3). We selected the miRNA candidates that met the following criteria: (i) fold change > 3 compared to NC; and (ii) normalized intensity of miRNA candidates > 100. miR-1275 was the only specific sponging miRNA for circCDYL (Fig. 4b). We then verified the results of miRNA microarray by qRT-PCR and found that miR-1275 was enriched by circCDYL probe (Fig. 4c). Similarly, miRNA pull-down assay showed that biotinylated miR-1275 also enriched circCDYL (Fig. 4d). In addition, we tested the basic expression of miR-1275 as well as other miRNAs that were known to be highly upregulated in breast cancer (including miR-7, miR-30c, miR-135b, miR-16, miR-206, miR-200a) [39, 40]. Expression of miR-1275 was similar to miR-30c, miR-135b and miR-7, and significantly higher than miR-200a and miR-206 in MDA-MB-231 cells (Fig. S6B). Further analysis from online website RNAhybrid 2.0 (https://bibiserv.cebitec.uni-bielefeld.de/rnahybrid/) showed that circCDYL had 3 binding sites of miR-1275 (Fig. S6C). To further confirm this result, we performed a dual luciferase reporter assay by co-transfecting miR-1275 mimic and the luciferase reporters into HEK-293 T cells. In the non-mutant groups, miR-1275 mimic reduced the luciferase reporter activity by 65%, and this result can be reversed after mutating either binding site 2 or 3 of circCDYL sequence in luciferase reporters, indicating that circCDYL bound to miR-1275 specifically at these two binding sites (Fig. 4e). In addition, the expression of miR-1275 remained unchanged after silencing of circCDYL in both MCF-7 and MDA-MB-231 cells, suggesting that circCDYL interacted with miR-1275 without affecting the expression of miR-1275 (Fig. S6D). In LC3-mCherry-GFP-labeled MDA-MB-231 cell, LC3 dots were increased after miR-1275 inhibitor transfection and significantly decreased after miR-1275 mimic transfection, suggesting miR-1275 promoted autophagic level (Fig. 4f, Fig. S6E). In MDA-MB-231 cells, LC3-II was up-regulated after miR-1275 inhibitor transfection and down-regulated after miR-1275 mimic transfection (Fig. 4g). Cell viability assays indicated that the proliferative speed was faster after miR-1275 inhibitor transfection and was slower after miR-1275 mimic treatment in both MCF-7 and MDA-MB-231 (Fig. S6F).

### circCDYL promotes autophagic flux via miR-1275 and is responsible for the proliferation of BC cells

We then tested whether circCDYL regulated autophagic level and proliferation via miR-1275 sponging mechanism. In MDA-MB-231 cells, Western Blot indicated that co-transfection with miR-1275 mimic and circCDYL over-expressing plasmid could restore the effect of LC3-II up-regulation by circCDYL overexpression, in contrast, co-transfection with miR-1275 inhibitor and circCDYL siRNA could restore the effect of LC3-II down-regulation via circCDYL silencing (Fig. 4h). In addition, in mCherry-GFP-LC3-labeled MDA-MB-231, we observed that co-transfection of miR-1275 mimic and circCDYL over-expressing plasmid partially restored the effect of circCDYL overexpression on the increased autophagic level, while co-transfection of miR-1275 inhibitor and circCDYL siRNA partially rescued this effect of circCDYL silencing (Fig. S6G). In addition, plate colony formation assay and cell viability assay showed that transfection of miR-1275 mimic also partially restored the proliferation-promoting effect after circCDYL overexpression in both MCF-7 and MDA-MB-231 cells, while addition of miR-1275 inhibitor partially rescued the effect of knocking down circCDYL (Fig. 4i-j).

### circCDYL regulates ATG7 and ULK1 expression via miR-1275 sponge

**Fig. 5:**
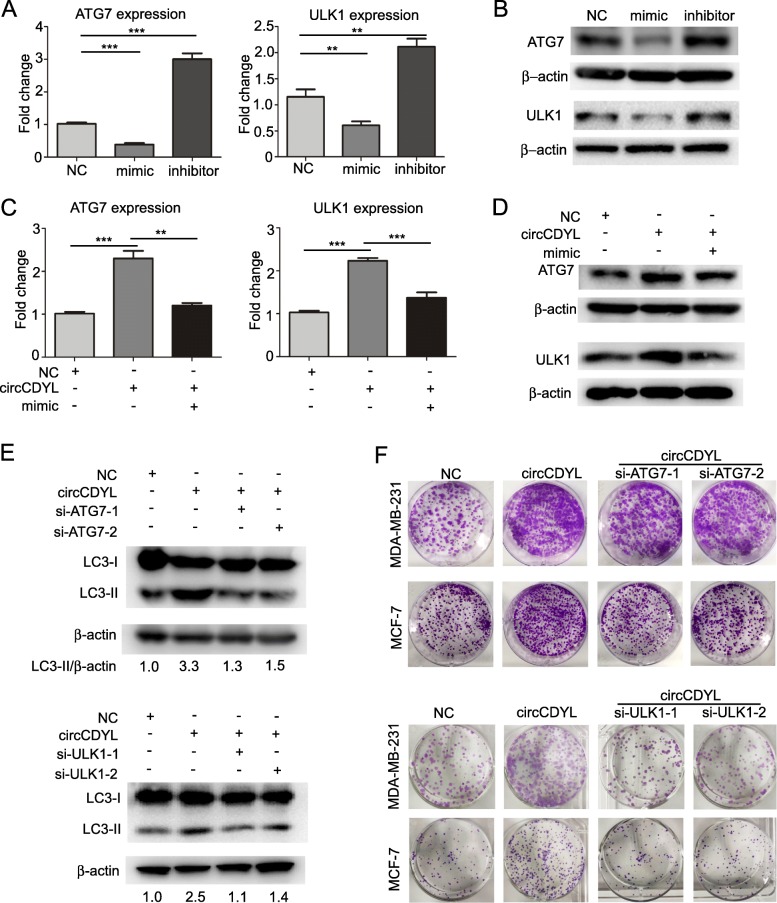
circCDYL promotes proliferation of breast cancer cells via miR-1275-ATG7/ ULK1-autophagic axis. **a** ATG7 and ULK1 expression of MDA-MB-231 after transfection with miR-1275 mimic or inhibitor, as detected by qRT-PCR. **b** ATG7 and ULK1 expression of MDA-MB-231 after transfection with miR-1275 mimic or inhibitor, as detected by Western Blot. **c**, **d** ATG7 and ULK1 expression in MDA-MB-231 after transfection with circCDYL over-expressing plasmid and ATG7 or ULK1 siRNA, as detected by qRT-PCR (C) and Western Blot (D). **e** Western Blot analysis of LC3-II expression in MDA-MB-231 after co-transfection with circCDYL over-expressing plasmids and ATG7 or ULK1 siRNA. **f** Proliferation level of MCF-7 and MDA-MB-231 after co-transfection with circCDYL over-expressing plasmids and ATG7 or ULK1 siRNA, as detected by plate colony formation

Three online websites, TargetScanHuman, miRNA.org and miRNAWalk, were used to predict the potential target genes of miR-1275. As shown in Fig. S7A, total seven autophagy-associated genes, including ULK1, ATG3, ATG4C, ATG4D, ATG7, ATG10, ATG14, were shown as the target genes of miR-1275 in at least two online websites**.** We further confirmed that miR-1275 mimic decreased ULK1 and ATG7 expression at RNA level by 55 and 63% respectively, while miR-1275 inhibitor increased ULK1 and ATG7 expression by 83 and 180% in MDA-MB-231 (Fig. 5a). However, ectopic expression of miR-1275 has no influence on the expression of ATG3, ATG4C, ATG4D, ATG10 and ATG14 (Fig. S7B). Western Blot analysis indicated that miR-1275 mimic decreased the protein level of ULK1 and ATG7, while miR-1275 inhibitor increased these proteins in MDA-MB-231 (Fig. 5b). Furthermore, miR-1275 pull-down assay showed that biotinylated miR-1275 mimic enriched ATG7 and ULK1 mRNA, suggesting that ATG7 and ULK1 are the target genes of miR-1275 (Fig. S7C). Moreover, we found that overexpressing circCDYL-wt significantly increased ATG7 and ULK1 expression, while the circCDYL-mu overexpressing had no effect on ATG7 and ULK1 regulation (Fig. S7D). Transfection of miR-1275 inhibitor could restore ATG7 and ULK1 expression after circCDYL knockdown, and transfection of miR-1275 mimic could restore ATG7 and ULK1 expression after circCDYL overexpression at both RNA and protein level (Fig. 5c, d). The results above indicate that circCDYL regulates the expression of ATG7 and ULK1 via miR-1275 sponge.

Next, we elucidated whether circCDYL regulates autophagy and proliferation of breast cancer cells via miR-1275-ATG7/ULK1 axis. By using siRNA, we successfully silenced ATG7 or ULK1 in both MCF-7 and MDA-MB-231 cells (Fig. S7E). Importantly, silencing of ATG7 or ULK1 restored the effect of circCDYL overexpression on increasing the autophagic marker LC3-II, as detected by Western Blot (Fig. 5e). In addition, silencing ATG7 restored the inhibitory effect of circCDYL overexpression on increasing autophagosomes in mCherry-GFP-labeled MDA-MB-231 cells, as determined by LC3 dots (Fig. S7F). In functional experiments, plate colony formation assay showed that the proliferation-promoting effect of circCDYL overexpression was attenuated after co-transfection of circCDYL overexpression plasmid with ATG7 or ULK1 siRNA (Fig. 5f).

In addition, we tested the Ki67, ATG7 and ULK1 by IHC, LC3 dots by IF and circCDYL expression by ISH in tumor tissues of Cohort 1 (Fig. S8A). The tumor tissues with higher CDYL expression exhibited a higher Ki67 index (Fig. S8B). Linear regression analysis showed the circCDYL expression was positively associated with ATG7 and ULK1 expression (Fig. S8C). Low ATG7 or ULK1 was associated with longer DFS among these patients (ULK1: HR 2.745, 95% CI 1.393-4.410, *P* = 0.0037; ATG7: HR 1.98, 95% CI 1.012–3.909, *P* = 0.0462, Fig. S8D).

### Linear CDYL has weak potential to act as miR-1275 sponge

We then examined whether circCDYL possesses more miR-1275 sponging power compared to linear CDYL via a series of experiments. Firstly, AGO2 RIP assays were employed and showed that AGO2 antibody enriched much more circCDYL than linear CDYL (circCDYL: power value =10.7*10^− 9^; linear CDYL: power value =2.2*10^− 9^) (Fig. 4a, Fig. S9A). Secondly, two luciferase reporter plasmids with total length of linear CDYL or circCDYL were conducted. Luciferase reporter assays showed that overexpression of miR-1275 mimic dramatically reduced the circCDYL luciferase reporter activity up to 65% but only reduced linear CDYL luciferase reporter activity by 25% (Fig. 4e and Fig. S9B). Furthermore, circCDYL pull-down and linear CDYL pull-down assay revealed that, compared with linear CDYL probes, circCDYL probe enriched much more miR-1275 (circCDYL probes: power value =5.9*10^− 10^; linear CDYL probes: power value =1.4*10^− 11^) (Fig. 4c and Fig. S9C). Moreover, the inhibitory effect of miR-1275 mimic on ATG7 and ULK1 expression could be only rescued by overexpressing circCDYL but not by linear CDYL overexpression in the MDA-MB-231 cells (Fig. S9D). Therefore, the results above provides further evidence that circCDYL possesses more miR-1275 sponging power compared to linear CDYL.

### circCDYL promotes proliferation via autophagy in breast cancer orthotopic animal model

**Fig. 6:**
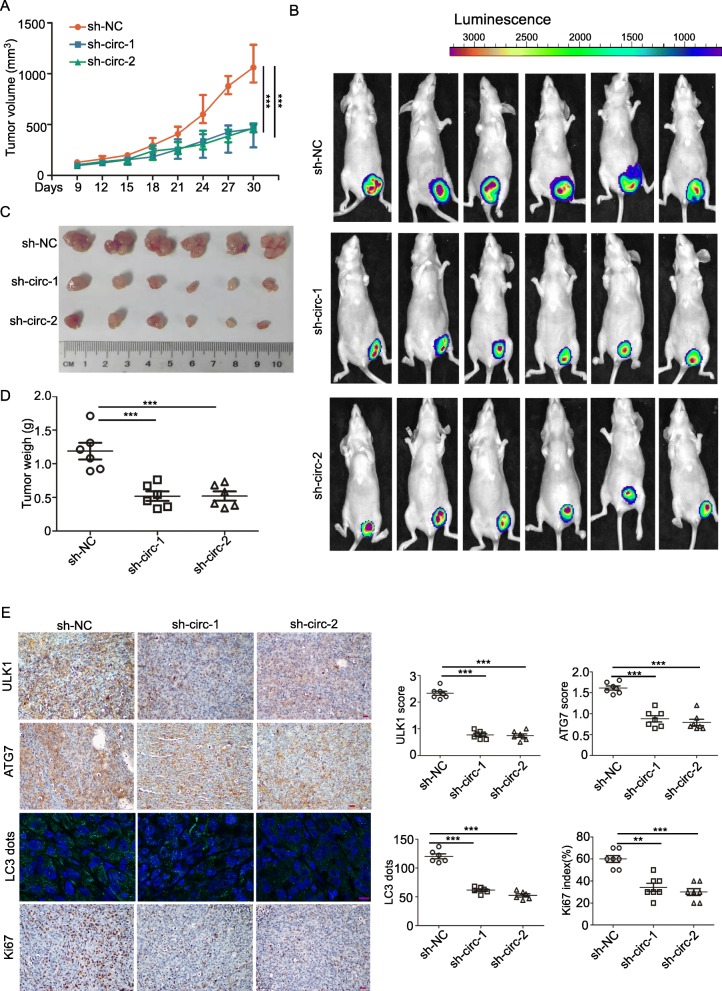
circCDYL promotes progression of breast cancer in vivo. **a** Tumor size derived from MDA-MB-231 cell line was measured in Balb/c nude mice with orthotopic tumors in the fourth left mammary fat pad. **b** Live imaging of the animals prior to euthanasia, and photon intensities are indicated above the picture. **c**, **d** Tumor size (C) and tumor weight (D) measured after tumor excision. **e** IHC staining of ATG7, ULK1 and Ki67, and IF detection of LC3 dots in orthotopic tumors derived from MDA-MB-231 cell line. Scale bar = 50 μm

To further confirm that circCDYL could promote BC cells proliferation in vivo, we established breast cancer orthotopic model in Balb/c nude mice. The tumors derived from MDA-MB-231 cells with stable knockdown of circCDYL (sh-circCDYL) had significantly slower growth than the control group (sh-NC) (Fig. 6a). The average tumor size of sh-circCDYL group was much smaller than sh-NC group as indicated by live imaging (Fig. 6b) or by measuring tumor sizes after tumor excision (Fig. 6c). sh-circCDYL group had a lighter weight than sh-NC group (Fig. 6d). In addition, the resected tumors were made into paraffin-embedded sections, followed by detection of ATG7, ULK1, LC3 dots and Ki67. sh-circCDYL group had lower expression of ATG7, ULK1, less LC3 dots and lower Ki67 index than sh-NC group (Fig. 6e). The results demonstrate that circCDYL plays an important role in promoting breast cancer progression in vivo via autophagy.

## Discussion

Breast cancer is the most common malignancies among women [1, 41]. Though breast cancer exhibits good prognosis, some breast cancer patients still suffer tumor progression. One of the emerging concepts to promote progression of breast cancer is autophagy [4, 8–11]. It is now known that the dysregulation of autophagy promotes progression of breast cancer. However, the molecular mechanism of autophagy in the progression of breast cancer is still unknown. In this study, we identified and characterized an autophagy associated circRNA circCDYL by deep sequencing in BC tissues with different autophagic level. Clinically, data from three independent cohorts showed the strong clinical relevance between circCDYL expression and prognosis as well as clinical response to therapy in breast cancer patients. Mechanistically, circCDYL accelerates autophagic flux via sponging miR-1275 and affects its downstream targeted gene ATG7 and ULK1, thus promotes the proliferation of breast cancer (Fig. S10). It is well known that ULK1 is key molecule in ULK1 complex, which is essential in the initiation of autophagy [42]. ATG7 acts as an E1-like activating enzyme to transform LC3-I to LC3-II by conjugation of LC3-I with phosphatidylethanolamine (PE) during autophagosome formation, and LC3-II plays an essential role in autophagosome formation [43]. All these suggest that circular circCDYL promotes proliferation of breast cancer via escalation of autophagic flux and plays an important role in the progression of breast cancer.

circRNAs are generally considered to be molecular flukes or byproducts of transcription. However, increasing evidences indicate that circRNAs are abundant in cells and play an important role in regulating disease progression [30]. Tantamount studies indicate that circRNAs work as miRNA sponge by trapping and halting the function of target miRNAs [7, 20–22]. Various studies indicate that circRNAs with intronic sequence may regulate transcription of parent genes in the nucleus [24, 25] and circRNAs with internal ribosome entry site (IREs) and open reading frame (ORF) could potentially be translated into proteins [26–28]. circCDYL, a circular RNA derived from the fourth exon of CDYL gene, is mainly located in cytoplasm and had no intronic sequence, IREs and ORF (online website: http://reprod.njmu.edu.cn/circrnadb, data not shown), suggesting that circCDYL might not locate in nucleus or translate into proteins. Emerging evidences shows that certain circRNAs can form a circRNAs-miRNA-AGO2 complex if the circRNA can work as miRNA sponge [22, 23, ﻿38﻿]. According to our data, AGO2 RIP experiment shows that circCDYL indeed bind to AGO2, confirming circCDYL’s role as a miRNA sponge. We used circRNAs pulldown experiments and miRNA microarray to verify the miRNA candidate of circCDYL. miRNAs pulldown and circRNA pulldown as well as dual-luciferase reporter assay demonstrated that circCDYL functions as miR-1275 sponge and has two binding sites for miR-1275. In addition to the discovery of circCDYL, to the best of our knowledge, this is the first report in elucidating the role of miR-1275 in breast cancer, and we are the first to prove that miR-1275 inhibits proliferation of breast cancer cells.

It is interesting that only circCDYL has the biological function on proliferation and autophagy regulation, while the linear form of CDYL do not possess the same function., although they have the same miR-1275 biding sites. Several reasons may account for this: (i) Our research indicates that miR-1275 sponge potential of circCDYL is much stronger than linear CDYL. (ii) The major function of linear CDYL is to work as mRNA and translate into protein, and CDYL protein may play different role in biological function. The mechanism function of linear CDYL is much more complex than circCDYL, and further researches are required.

Identifying new molecular biomarkers for diagnosis and predicting prognosis and clinical efficacy of breast cancer patients is of high clinical significance. In this current study, we found that circCDYL expression in both breast cancer patients’ serum and tissue were much more abundant than those from the normal donors and adjacent normal tissues, suggesting that circCDYL could be utilized as a molecule for breast cancer diagnosis and therapy. Second, clinical data showed that increased circCDYL in breast cancer tissues was associated with more advance clinical stage, more metastasis and shorter survival, indicating that circCDYL could be a potential molecule for predicting breast cancer prognosis. Besides, the expression level of circCDYL in breast cancer tissues was positively associated with the level of LC3 dots, indicating that circCDYL could be a marker reflecting autophagic level of cancer.

Interestingly, we observed a real time kinetics of serum circCDYL in MBC patients and found that serum circCDYL level was decreased in patients who responded well to chemotherapy, indicating that serum circCDYL level might be a potential molecule to predict the clinical response to anti-cancer therapy. Several hypotheses are proposed to address this clinical relevance. (i) The abundance of circCDYL in cancer tissue were released into circulation as necrosis occurs due to ischemic and hypoxic tumor microenvironment, which could lead the induction of autophagy. We found that circCDYL expression was significantly increased in BC cells after hypoxia or starvation treatment, suggesting that more circCDYL were released into circulation when tumor cells are in hypoxic or ischemic condition. (ii) Circulating tumor cells (CTCs) bring circCDYL into the circulation, which could be a reservoir for circCDYL molecules. MBC patients are reported to have more CTCs [44] and our study found that MBC patients had more serum circCDYL than EBC patients, indirectly validating this hypothesis. (iii) Breast cancer cells release circCDYL through exosomes secretion. One research indicated that lung cancer cells can secret exosomal circCDYL and release into circulation [45], indicating that this pathway might be true for breast cancer as well. Furthermore, confirming the expression of circRNA biomarkers by minimally invasive liquid biopsies allows the clinician to monitor breast cancer progression and response to treatment in real-time setting, which would significantly improve the development of earlier intervention and to develop more precise therapy strategies that tailors to individual needs.

## Conclusion

In summary, we identified an autophagy-associated circRNA (circCDYL) and demonstrated its strong correlations with autophagy in breast cancer, as well as its prognostic and predictive value in breast cancer patients. Mechanistically, we proved that circCDYL regulates proliferation of breast cancer cells via miR-1275-ATG7/ULK1-autophagic axis. circCDYL could be used as a novel circRNA biomarker for predicting the prognosis and clinical response to therapy as well as a potential therapeutic target, which provide real-time information for decisions making in personalized treatments.

## Supplementary information

**Additional file 1.** Supplementary Materials and Method. **Figure S1.** LC3 expression was associated with poor survival of breast cancer. **Figure S2.** Autophagy induction model in vitro. **Figure S3.** Clinical significance of linear CDYL in BC. **Figure S4.** The functional role of circCDYL in BC cell lines. **Figure S5.** The functional role of linear CDYL in BC cell lines. **Figure S6.** circCDYL works as a sponge for miR-1275. **Figure S7.** miR-1275 targets 3’ UTR of ATG7 mRNA. **Figure S8.** ATG7 and ULK1 protein expression in the SYSMH Cohort 1 with 113 breast cancer patients. **Figure S9.** The miR-1275 binding power of linear CDYL. **Figure S10.** Graphic abstract. **Table S1.** circRNAs deep sequencing in breast cancer tissue with different autophagic level. **Table S2.** Patient characteristics stratified by expression of linear CDYL.** Table S3.** miRNAs microarray after circRNA pull down. **Table S4.** Sequence of siRNA or shRNA used in current study. **Table S5.** Primers used in current study. **Table S6.** Probes used in current study.

## Data Availability

The datasets obtained and analyzed during the current study were made available from the corresponding authors through request.

## Abbreviations

95% CI: 95% confidence interval
circCDYL: Autophagy-associated circRNA
circRNA: circular RNA
ddPCR: droplet digital PCR
EBC: Early breast cancer
ER: Estrogen receptor
HR: Hazard ratio
*i.p*: intra-peritoneally injection
IF: Immunofluorescence
IHC: Immunohistochemical staining
MBC: Metastatic breast cancer
NC: Negative control
qRT-PCR: Real-Time Quantitative Reverse Transcription PCR
SYSMH: Sun Yat-sen Memorial Hospital
