# Supplementary material for: Autophagy-associated circRNA circCDYL augments autophagy and promotes breast cancer progression
Source: Mol Cancer. 2020 Mar 25;19:65. doi: 10.1186/s12943-020-01152-2 (PMC7093993; doi:10.1186/s12943-020-01152-2)
Supplement: Supplementary file 1 — Additional file 1. Supplementary Materials and Method. Figure S1. LC3 expression was associated with poor survival of breast cancer. Figure S2. Autophagy induction model in vitro. Figure S3. Clinical significance of linear CDYL in BC. Figure S4. The functional role of circCDYL in BC cell lines. Figure S5. The functional role of linear CDYL in BC cell lines. Figure S6. circCDYL works as a sponge for miR-1275. Figure S7. miR-1275 targets 3’ UTR of ATG7 mRNA. Figure S8. ATG7 and ULK1 protein expression in the SYSMH Cohort 1 with 113 breast cancer patients. Figure S9. The miR-1275 binding power of linear CDYL. Figure S10. Graphic abstract. Table S1. circRNAs deep sequencing in breast cancer tissue with different autophagic level. Table S2. Patient characteristics stratified by expression of linear CDYL. Table S3. miRNAs microarray after circRNA pull down. Table S4. Sequence of siRNA or shRNA used in current study. Table S5. Primers used in current study. Table S6. Probes used in current study. [file 12943_2020_1152_MOESM1_ESM.doc]

**Supplementary Data**

**Title:** Autophagy-associated circRNA circCDYL augments autophagy and promotes breast cancer progression.

Gehao Liang^1^,*, Yun Ling^1^,*, Maryam Mehrpour^2,3^, Phei Er Saw^4^, Zihao Liu^1^, Weige Tan^5^, Zhenluan Tian^1^, Wenjing Zhong^1^, Wanyi Lin^1^, Qing Luo^1^, Qun Lin^1^, Qiufang Li^1^, You Zhou^6,7^, Ahmed Hamai^2,3^, Patrice Codogno^2,3^, Jun Li^8^, Erwei Song^1,9,10^, Chang Gong^1,10,#^.

^1^Breast Tumor Center, Guangdong Provincial Key Laboratory of Malignant Tumor Epigenetics and Gene Regulation, Sun Yat-sen Memorial Hospital, Sun Yat-sen University, Guangzhou, 510120, China;

^2^Institut Necker-Enfants Malades (INEM), Inserm U1151-CNRS UMR 8253, Paris, 75993, France;

^3^Université Paris Descartes-Sorbonne Paris Cité, Paris, 75993, France.

^4^Medical Research Center, Guangdong Provincial Key Laboratory of Malignant Tumor Epigenetics and Gene Regulation, Sun Yat-sen Memorial Hospital, Sun Yat-sen University, Guangzhou, 510120, China;

^5^Breast surgery department, the First Affiliated Hospital of Guangzhou Medical University, Guangzhou Medical University, Guangzhou, 510120, China;

^6^Systems Immunity University Research Institute and Division of Infection and Immunity, School of Medicine, Cardiff University, Cardiff, CF14 4XN, UK;

^7^Minerva Foundation Institute for Medical Research, Helsinki, 00290, Finland;

^8^Department of Biochemistry, Zhongshan School of Medicine, Sun Yat-sen University, Guangzhou, 510080, China;

^9^Program of Molecular Medicine, Zhongshan School of Medicine, Sun Yat-Sen University, Guangzhou, 510080, China;

^10^Guangzhou Regenerative Medicine and Health Guangdong Laboratory, Guangzhou, 510005, China.

*Gehao Liang and Yun Ling contributed equally to this study.

**Correspondence:** Chang Gong, Breast Tumor Center, Sun Yat-sen Memorial Hospital, Sun Yat-sen University, 107 Yanjiang West Road, Guangzhou, 510120, China, Phone: +86-20-81333407, Fax: +86-20-81333407; Email: [changgong282@163.com](mailto:changgong282@163.com) or [gchang@mail.sysu.edu.cn](mailto:gchang@mail.sysu.edu.cn).

**Inventory**

Supplementary Materials and Methods

10 Supplementary Figures

6 Supplementary Tables

**Supplementary Materials and Methods**

**Cell culture and transfections**

MDA-MB-231 and MCF-7 cell lines were purchased from ATCC, and HEK-293T cell line was a gift from the Department of Hepatobiliary Surgery at the Sun Yat-sen Memorial Hospital (Guangzhou, China). MDA-MB-231 and HEK-293T were cultured in DMEM (Invitrogen, Carlsbad, USA) supplemented with 10% FBS, and MCF-7 was cultured in RPMI 1640 (Invitrogen, Carlsbad, USA) supplemented with 10% FBS. For transient transfection, 3 pmol siRNA (siRNA sequence shown in **Table S4**) or 125 ng circCDYL over-expressing plasmid was added to cells with 3.75 ul [lipofectamine 3000](https://www.sogou.com/link?url=DSOYnZeCC_owkDvmYG0gMz-JrNZwwuWK1VqBoQ46pYN0gohKX14THasYM9mIOXNn2uFhv1mjiPiuLaNMGv04nw..) (Invitrogen, Carlsbad, USA). After 48 hours, total RNA or protein were collected two days after transfection. Stable mCherry-GFP-LC3 MDA-MB-231, sh-circCDYL and overexpressed-circCDYL MDA-MB-231 cell lines were conducted by infecting respectively with mCherry-GFP-LC3 lentivirus (Hanbio, Shanghai, China), sh-circCDYL and overexpressed-circCDYL lentivirus (Hanbio, Shanghai, China), followed by a selection with 2 mg/ml puromycin for 2 weeks. As for autophagy induction, cancer cells were cultured under 0.2% O_2_ hypoxic condition for 24 and 48 hours, or treated under starvation condition with Earle's Balanced Salt Solution (EBSS, Cat Lot 14155063, Gibco) for 2, 4, 6 hours[1, 2].

**Plate colony formation assay**

After relative treatment, a total of 1000 MDA-MB-231 or MCF-7 cells were seeded in 6-well plates and were cultured at 5% CO_2_ 37° C for 10 days. The cells were fixed with 4% formaldehyde for 10 mins, stained with 1% crystal violet for 30 mins. The colony numbers were calculated using Image J software.

**Cell vitality assay**

A total of 2 X 10^4^ MDA-MB-231 or MCF-7 cells after treatment were seeded in 12-well plates. In the continuous 5 days, cells were digested by 1ml trypsin, and resuspended thoroughly. 20 μl cell suspension was added into cell vitality plate (Ruiyu Bio-science, Shanghai, China), and the cell vitality was measured with an automatic cell counter (Countstar Bio-tech, IC100, Shanghai, China).

**RNA isolation, qRT-PCR and ddPCR**

The total RNA of BC cells was collected using TRIzol reagent (Invitrogen, Carlsbad, USA) and total RNA of 250 μl plasma were processed with HiPure Blood/Liquid RNA kit (Magen, R4163-02, Guangzhou, China). The RNA sample was reverse transcribed to cDNA using RT SuperMix (Vazyme Biotech, China) according to the manufacturer’s protocol. The real-time quantitative polymerase chain reaction (qRT-PCR) was performed using SYBR qRT-PCR Master Mix (Vazyme Biotech, China) on Roche LightCycler 480 II system (Roche, Switzerland) following the manufacturer’s instructions. ddPCR was constructed on the Nacia Crystal System (Sapphire Chips, Stilla Technologies, France) using EvaGreen (Cat#31000-50, HilisBio, China) and qPCR ThoughMix (Cat#95138-250, QuantaBio, USA). The advantage of ddPCR is able to detect the absolute copies of circRNA in serum[3, 4].The sequences of all primers were shown in **Table S5**.

**Western Blot**

Cells were lysed in RIPA lysis buffer (CW2333S, Cwbio, China). The concentration of protein was detected by bicinchoninic acid (BCA) kit (Lot 30342, Cwbio, China). In the process of electrophoresis, 30 μg protein was added into each well of the 12% SDS-PAGE. The protein in the gels were then transferred onto a PVDF membrane (Millipore, Schwalbach, Germany). The membranes were blocked with 5% non-fat milk at room temperature for 2 hours. Primary antibody anti-β-actin (#8457, Cell Signaling Technology, 1:1000), anti-ATG7 (GTX32459, Genetex, 1:1000), anti-ULK1 (A8529, Abclonal, 1:1000), anti-LC3 (L7543, [Sigma-Aldrich](https://www.sogou.com/link?url=DSOYnZeCC_qbflwkCr5PFyrio2RH5hH032GD08viWW4.), 1:1000), or anti-p62 (A0682, Abclonal, 1:1000) were respectively added and incubated at 4 ℃ overnight. Secondary antibody (Anti-Rabbit:7074S, Anti-Mouse:7076S, Cell Signaling Technology, 1:3000) was added and incubated for 2 hours at room temperature. Finally, the blots were detected by enhanced chemiluminescence kit (P90719, Millipore, USA) and was analyzed by Image Lab Software.

**RNA-binding protein immunoprecipitation (RIP)**

The RIP assay was performed by Magna RIP Kit (Millipore, USA) according to manufacturer’s instructions. Briefly, HEK-293T cells were fixed by 1% formaldehyde and lysed as described in the manufacturer's instructions. The lysates were first co-incubated with beads coated with either anti-AGO2 antibody (03-110, Millipore, USA) or negative control IgG (Millipore, USA) at 4°C overnight. The immunocomplex were then incubated with proteinase K for protein digestion. Total RNA of the immunocomplex was isolated for circCDYL and linear CDYL expression measurement by qRT-PCR.

**miRNA pull-down assay**

miRNA pulldown experiment was done according to previous literature[5]. Briefly, MDA-MB-231 cells were transfected with biotinylated miR-1275 mimic at a concentration of 20 nM. After 24 hours, cells were collected and fixed with 3% formaldehyde for 30 min prior to lysis by co-IP buffer. The mixture was then sonicated and centrifuged. C1 streptavidin magnetic beads was then added to the supernatant. Finally, the total RNA was extracted from the supernatant followed by qRT-PCR detection of ATG7, ULK1, linear CDYL and circCDYL.

**circRNAs deep sequencing and miRNAs microarray**

Total RNA was extracted from BC tissues with more- (n=5) and less- (n=5) LC3 dots by Trizol (Invitrogen, USA). Ribosomal and linear RNA were removed using Epicenter Ribo-Zero rRNA Removal Kit (Illumina, CA, USA) and RNAse R (Epicenter, CA, USA) respectively. Subsequently, RNA-seq libraries were prepared by using the NEBNext® Ultra™ RNA Library Prep Kit, and subjected to deep sequencing with an Illumina HiSeq 3000 at RiboBio Co. Ltd (Guangzhou, China). For miRNA microarray, RNA samples purified by circRNAs pull-down assay was sent to Kangchen Bio-tech (Shanghai, China) for miRNAs microarray analysis.

***In situ* hybridization (ISH)**

circCDYL and linear CDYL expression was detected in paraffin-embedded sections from BC tissues following protocols described in previous literature[6]. Digoxin-labeled circCDYL or linear CDYL probe was designed and synthesized by Synbio-Tech Company (Guangzhou, China). Briefly, after dewaxing and rehydration, the sections were digested with pepsin, hybridized with the digoxin-labeled circCDYL or linear CDYL probe at 37°C overnight. Subsequently the sections were incubated overnight at 4℃ with anti-digoxin antibody. Then the sections were stained with nitro blue tetrazolium/5-bromo-4-chloro-3-indolylphosphate. We determined the staining scores based on both the intensity and proportion of circCDYL or linear CDYL in the whole section. Intensity were recorded on a scale of 0 (no staining), 1 (light purple), 2 (purpleblue), and 3 (dark purple). Total score of expression = ∑proportion * intensity score.

**Fluorescence *in situ* hybridization (FISH)**

Cy3-labeled oligonucleotide probe for circCDYL and FAM-labeled oligonucleotide probe for miR-1275 were used for circRNA FISH. These probes were designed and synthesized by Synbio-Tech Company (Guangzhou, China) and the sequence of the probes are provided in the Supplementary Information (**Table S6**). Briefly, cells were seeded in a glass-bottom dish overnight and incubated with pre-hybridization solution at 37℃ for 30 mins. 20 μM of probes was dissolved in hybridization solution and added to slides or dish individually and hybridized overnight. After washing (4× SSC (saline sodium citrate) in 0.1% Tween-20 three times, 2 ×SSC once, and 1× SSC once, 5 min each wash), the slides were incubated with DAPI for 10 min at room temperature. Finally, they were covered with coverslip and observed under confocal microscope.

**Immunohistochemistry (IHC)**

IHC was performed on paraffin sections of breast cancer (BC) tissues or animal tumors using SP Rabbit & Mouse HRP Kit (Cwbio, China) according to manufacturer’s protocol. Primary antibodies against LC3 (1:100, L7543, [Sigma-Aldrich](https://www.sogou.com/link?url=DSOYnZeCC_qbflwkCr5PFyrio2RH5hH032GD08viWW4.)), ATG7(1:100, A0691, ABclonal), ULK1 (1:100, A8529, Abclonal) and Ki67 (1:100, GTX20833, Genetex) was used. Staining scores were determined based on both the intensity and proportion in the whole section, as described previously[7]. Intensity were recorded on a scale of 0 (no staining), 1 (light yellow), 2 (yellow), and 3 (dark yellow). Total score of detected protein = ∑proportion * intensity score.

**Immunofluorescence (IF)**

IF was done on paraffin sections of human BC tissues or animal experiments according to the protocols in previous literature[6] to detect LC3 dots. Briefly, the sections were incubated with primary antibodies against LC3 (1:100, L7543, Sigma-Aldrich) overnight at 4℃ after standard dewaxing process and incubated with fluorescein secondary antibody (FI-1000-1.5, Vector, Germany) for 2 hours at room temperature. Then they were incubated with DAPI for 10 mins at room temperature. Finally, the sections were covered with coverslip and observed under confocal microscope. The average number of autophagic dots in each field were calculated after observing 5 random fields of the section.

**References:**

1. Lefort S, Joffre C, Kieffer Y, Givel A, Bourachot B, Zago G, Bieche I, Dubois T, Meseure D, Vincent-Salomon A, et al: **Inhibition of autophagy as a new means of improving chemotherapy efficiency in high-LC3B triple-negative breast cancers.** *Autophagy* 2014, **10:**2122-2142.

2. Zheng Y, Chen Z, Gu Z, Yang X, Yu M, Zhao C, Lin J, Xu P, Zhu L, Jacob TJC, et al: **Starvation-induced autophagy is up-regulated via ROS-mediated ClC-3 chloride channel activation in the nasopharyngeal carcinoma cell line CNE-2Z.** *Biochem J* 2019, **476:**1323-1333.

3. Hindson CM, Chevillet JR, Briggs HA, Gallichotte EN, Ruf IK, Hindson BJ, Vessella RL, Tewari M: **Absolute quantification by droplet digital PCR versus analog real-time PCR.** *Nat Methods* 2013, **10:**1003-1005.

4. Li T, Shao Y, Fu L, Xie Y, Zhu L, Sun W, Yu R, Xiao B, Guo J: **Plasma circular RNA profiling of patients with gastric cancer and their droplet digital RT-PCR detection.** *Journal of Molecular Medicine* 2018, **96:**85-96.

5. Selimoglu-Buet D, Riviere J, Ghamlouch H, Bencheikh L, Lacout C, Morabito M, Diop M, Meurice G, Breckler M, Chauveau A, et al: **A miR-150/TET3 pathway regulates the generation of mouse and human non-classical monocyte subset.** *Nat Commun* 2018, **9:**5455.

6. Liu B, Sun L, Liu Q, Gong C, Yao Y, Lv X, Lin L, Yao H, Su F, Li D, et al: **A cytoplasmic NF-kappaB interacting long noncoding RNA blocks IkappaB phosphorylation and suppresses breast cancer metastasis.** *Cancer Cell* 2015, **27:**370-381.

7. Liu Z, Zhou Y, Liang G, Ling Y, Tan W, Tan L, Andrews R, Zhong W, Zhang X, Song E, Gong C: **Circular RNA hsa_circ_001783 regulates breast cancer progression via sponging miR-200c-3p.** *Cell Death Dis* 2019, **10:**55.


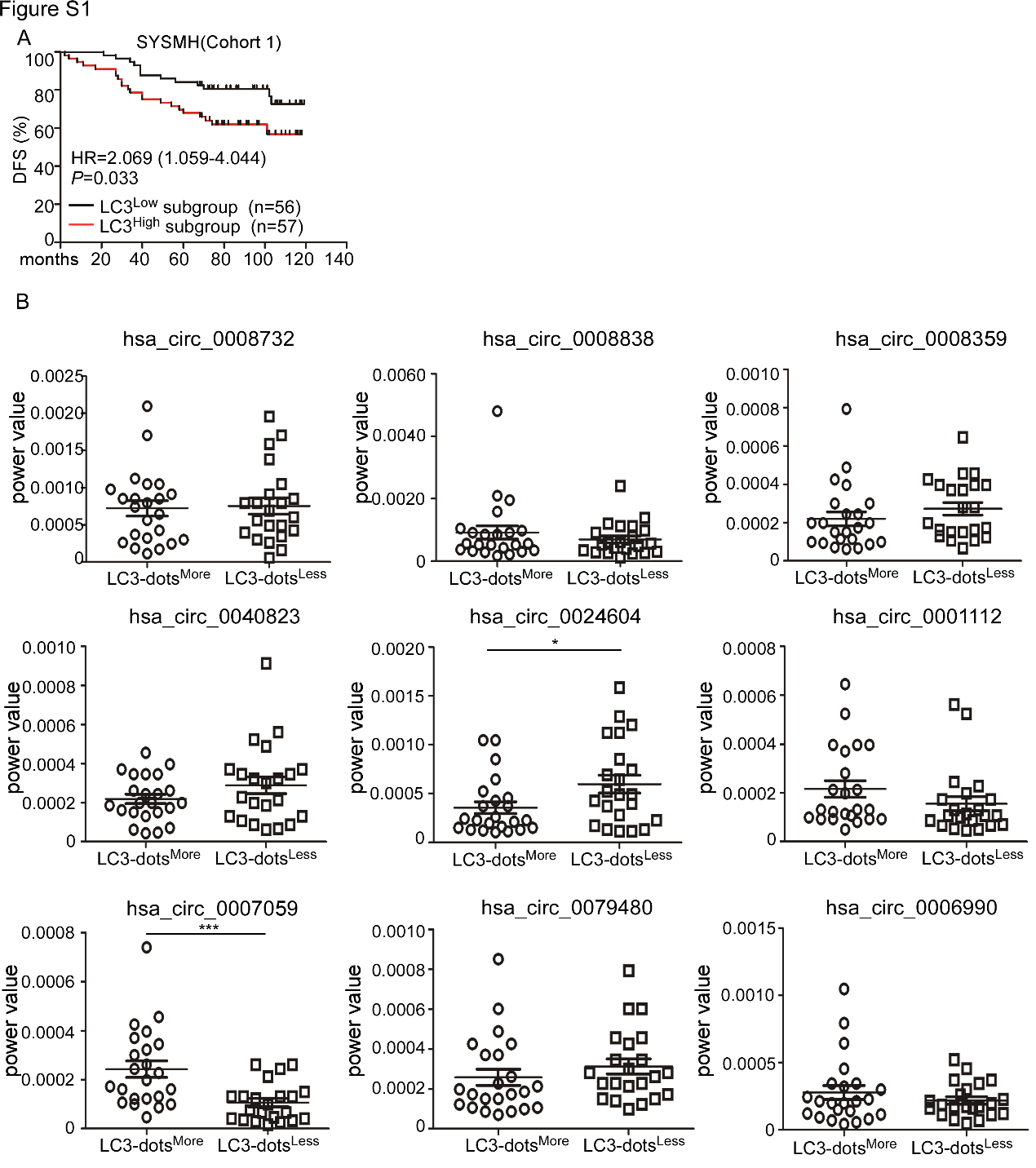
**Supplementary Figure**

**Fig S1. LC3 expression was associated with poor survival of breast cancer.** **A.** Autophagy marker LC3 (detected by IHC) was associated with DFS of BC in Cohort 1 from SYSMH (n=113). *DFS, disease free survival.* **B.** Expression of nine circRNAs as detected by qRT-PCR in LC3-dots^More^ (n = 23) and LC3-dots^Less^ (n = 22) BC tissues.


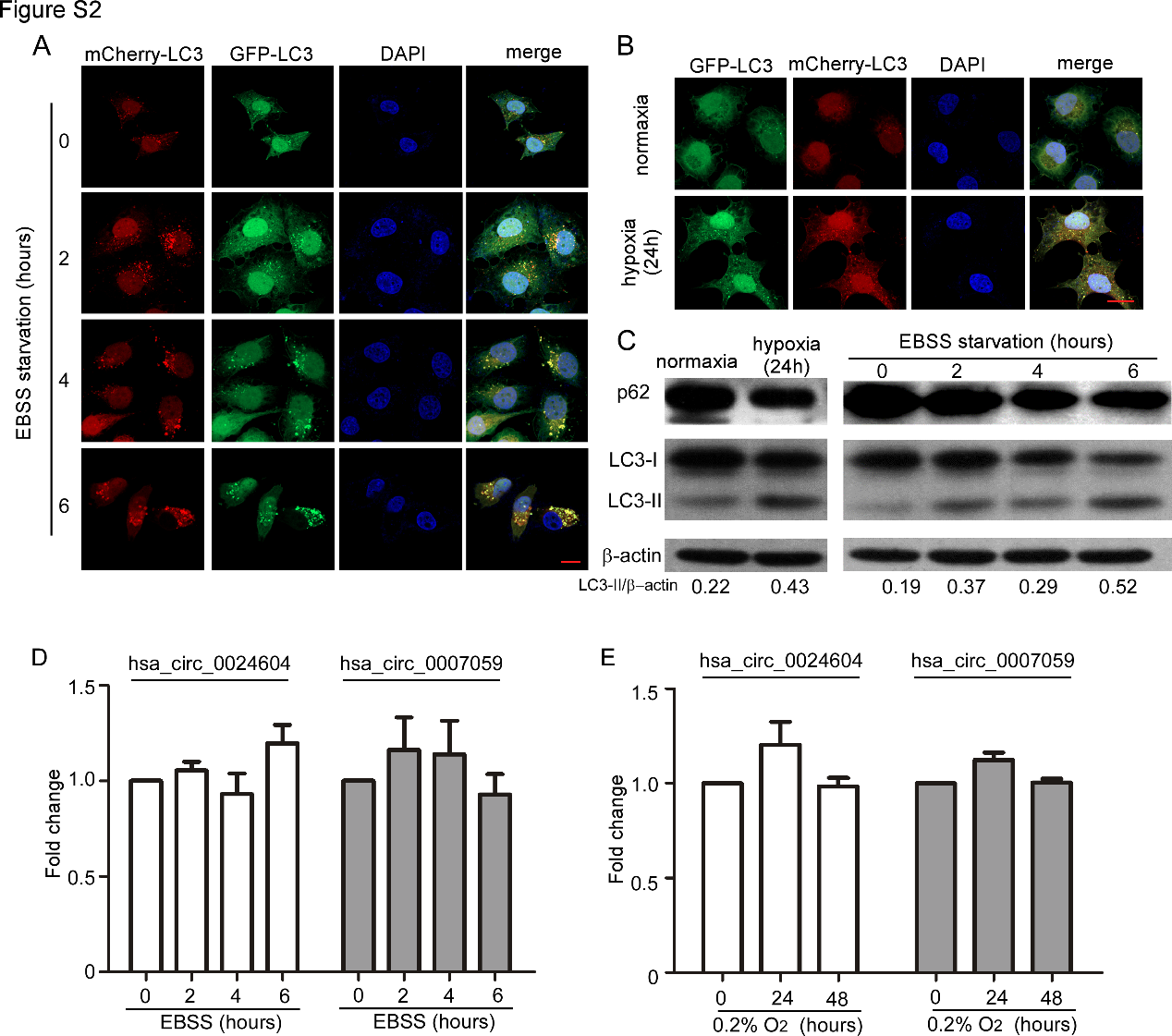
**Fig S2. Autophagy induction model *in vitro.* A-B.** Autophagosomes in mCherry-GFP-LC3 labeled MDA-MB-231 under EBSS at various time points (0, 2, 4, 6 hours) (A) and hypoxic induction (0.2% O_2_) at 0 and 24 hours (B), as detected by confocal microscope. Scale bar = 30μm. **C**. LC3 and p62 protein level in MDA-MB-231 under EBSS starvation at various time points (0, 2, 4, 6 hours) or under 0.2% O_2_ hypoxia induction at 0 and 24 hours, as detected by Western Blot. **D-E**. The relative expression of hsa_circ_0024604 and hsa_circ_0007059 under EBSS starvation induction at various time points (0, 2, 4, 6 hours) (D) and hypoxic induction (0.2% O_2_) at various time points (0, 24, 48 hours) (E) in MDA-MB-231 cells.


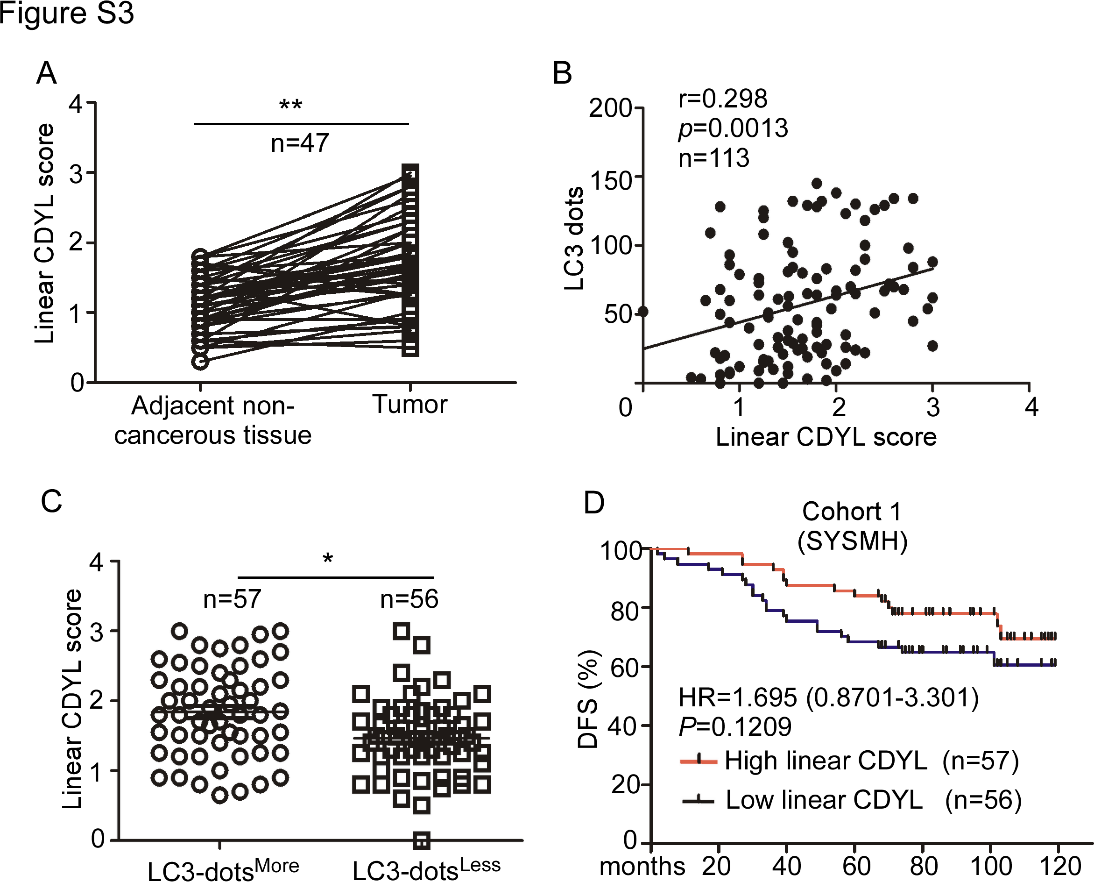
**Fig S3. Clinical significance of linear CDYL in BC. A.** Comparison of circCDYL expression between BC tissues and paired adjacent non-cancerous tissues by qRT-PCR (n = 47). **B.** Correlations between the expression of linear CDYL and the number of LC3 dots in tumors, analyzed by Pearson analysis. **C.** Quantitative analysis of linear CDYL expression in LC3-dots^More^ (n= 57) and LC3-dots^Less^ (n =56) BC tissues. **D.** Kaplan-Meier analysis of the correlation between linear CDYL expression and disease-free survival (DFS). **P* < 0.05, ***P* < 0.01, ****P* < 0.005. Error bars indicate S.E.M.

**
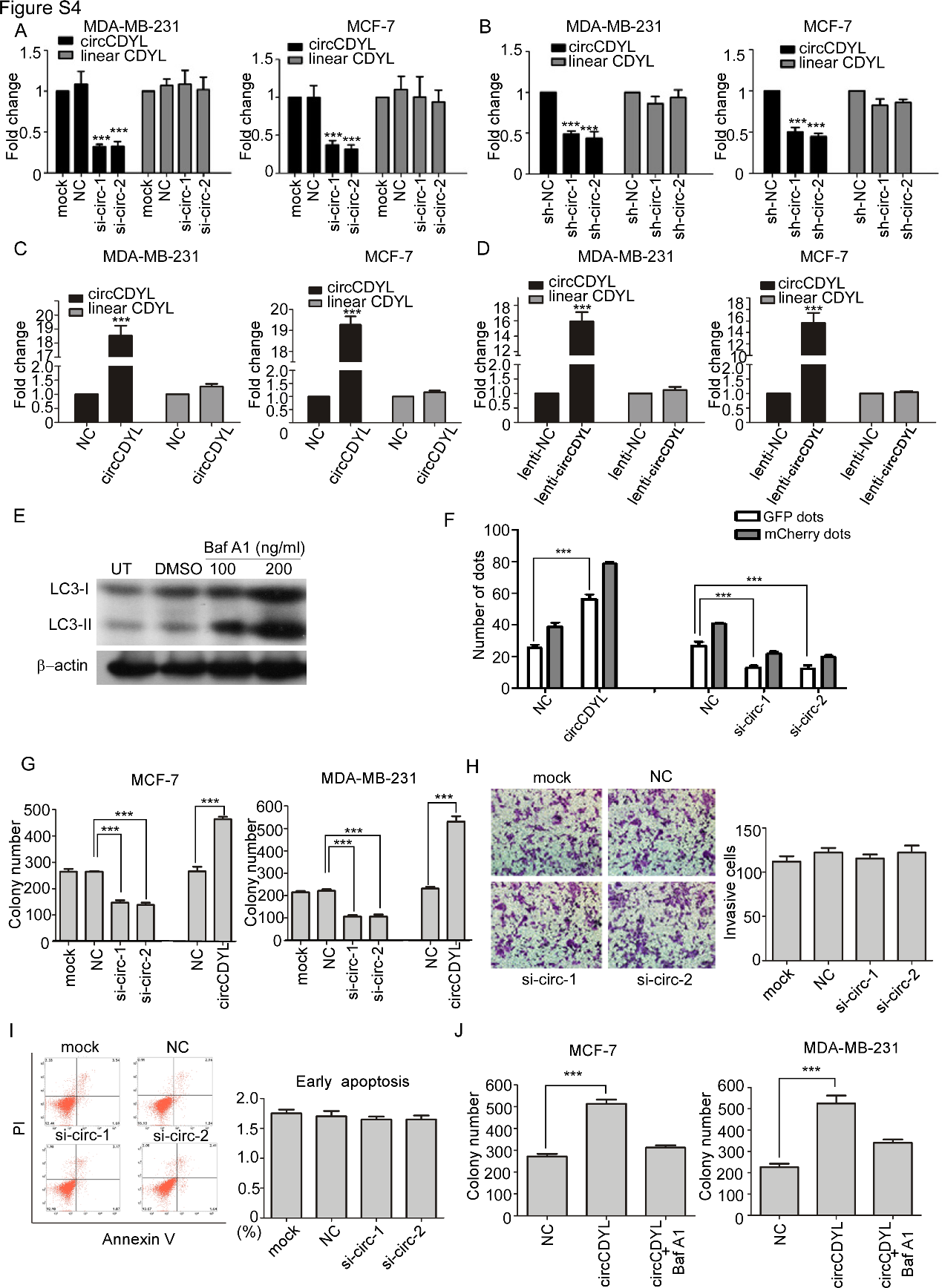
Fig S4. The functional role of circCDYL in BC cell lines. A, B.** qRT-PCR analysis of circCDYL and linear CDYL RNA in both MDA-MB-231 and MCF-7 after circCDYL silencing by circCDYL specific siRNA (A) and specific shRNA lentivirus (B). **C, D**. qRT-PCR analysis of circCDYL and linear CDYL RNA in both MDA-MB-231 and MCF-7 after circCDYL over-expression by circCDYL over-expressing plasmid (C) and circCDYL over-expressing lentivirus (D). **E.** LC3-II expression in MDA-MB-231 after autophagy inhibition by Bafilomycin A1, as detected by Western Blot. **F.** The quantification of LC3 dots in Fig 3C. **G.** The quantification of colonies in Fig 3E. **H, I.** Invasion assay (H) and PI/Annexin V double-staining analysis of apoptosis (I) in MDA-MB-231 cell line after circCDYL silencing
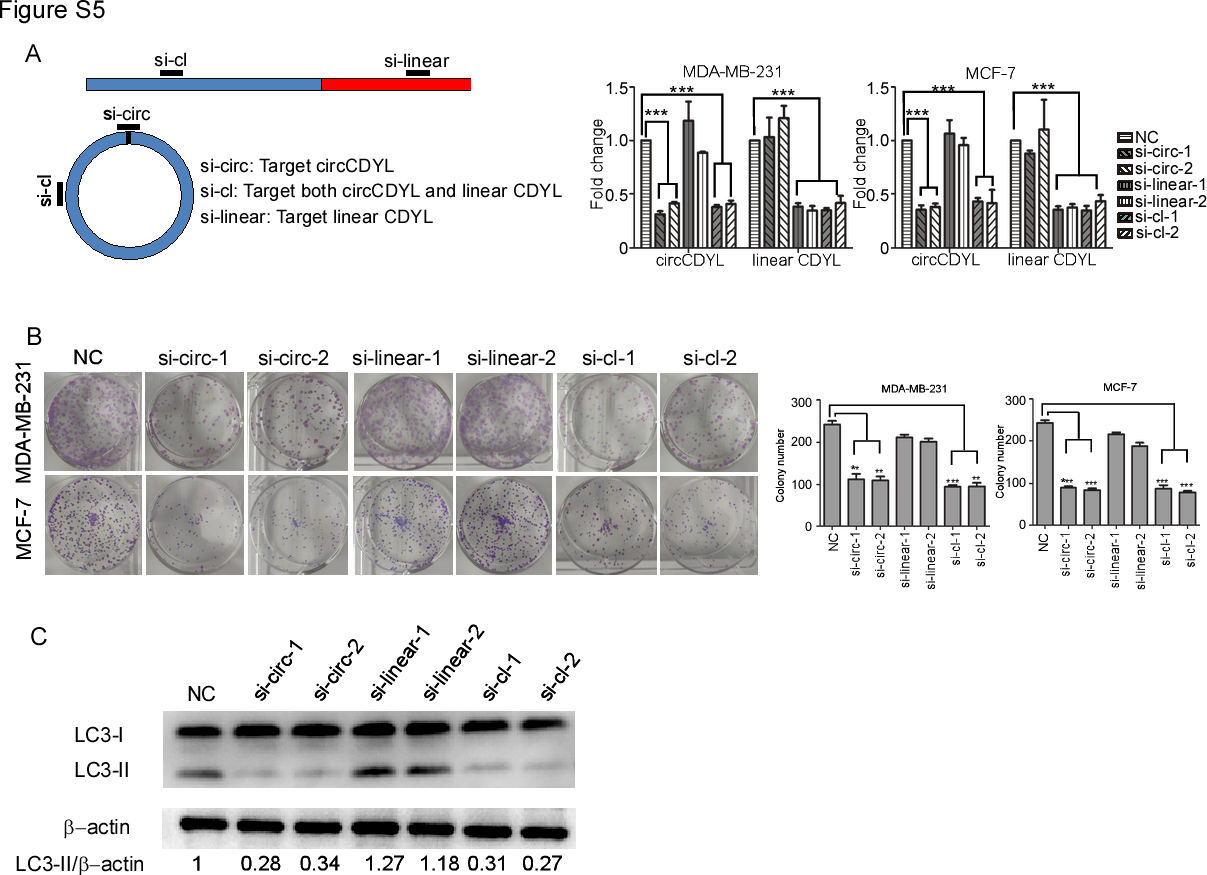
by circCDYL siRNA. **J.** The quantification of colonies in Fig 3F.

**Fig S5. The functional role of linear CDYL in BC cell lines. A.** Target sites of siRNA on linear CDYL or circCDYL (LEFT), qRT-PCR analysis of circCDYL and linear CDYL in both MDA-MB-231 and MCF-7 after treatment with three types of siRNAs (RIGHT). **B.** Plate colony formation assay with or without siRNA treatment. **C.** LC3-II expression in MDA-MB-231 after treatment with siRNA, as detected by Western Blot.


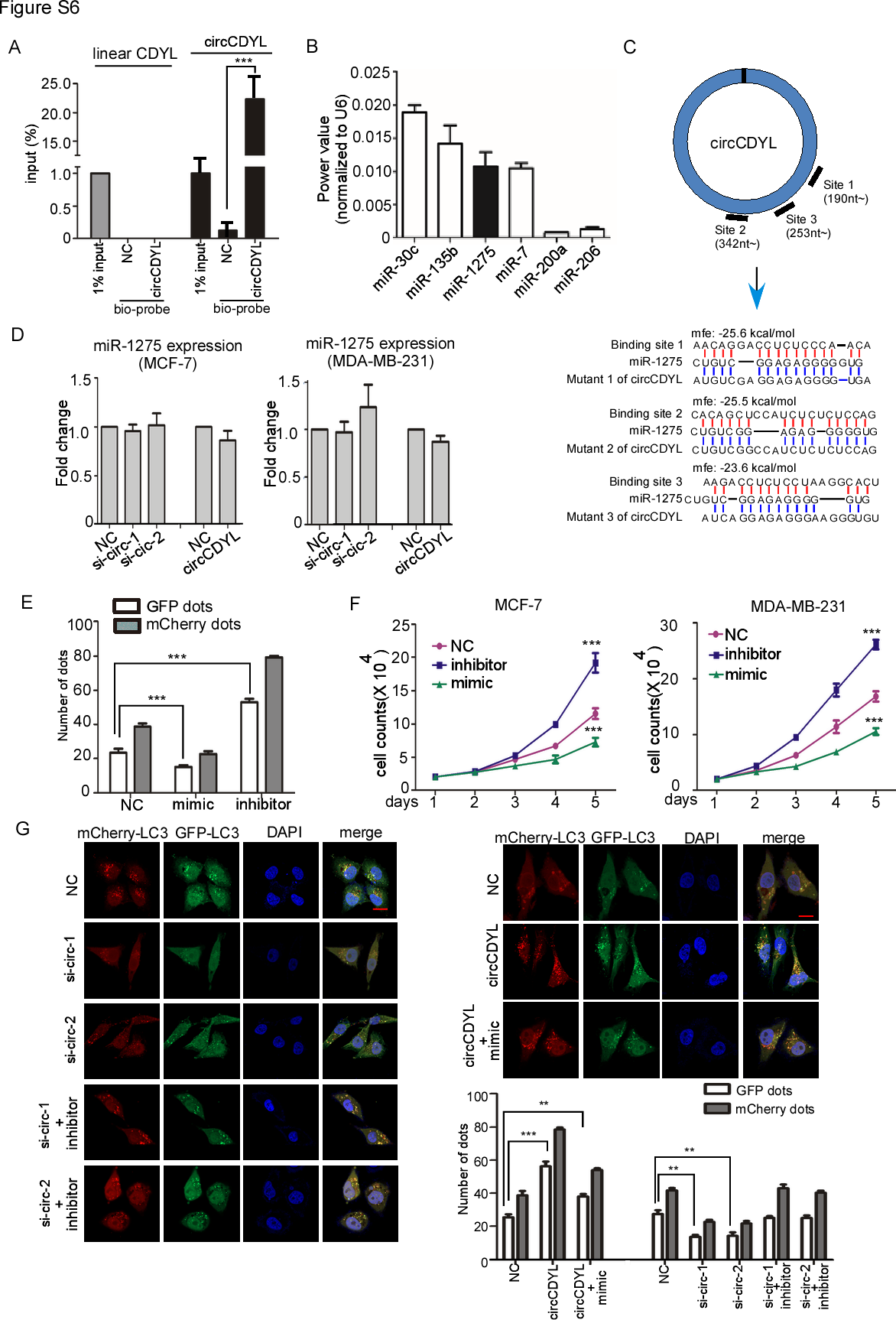
**Fig S6. circCDYL works as a sponge for miR-1275.** **A.** circRNA pull-down assay with NC or circCDYL biotinylated probe and followed by qRT-PCR detection of circCDYL and linear CDYL. **B.** Expression of miR-1275 and other miRNAs that were known to be highly upregulated in breast cancer (including miR-7, miR-30c, miR-135b, miR-16, miR-206, miR-200a), as detected by qRT-PCR. **C.** Three miR-1275 binding sites of circCDYL predicted by RNAhybrid online website, and the mutant sequence of these 3 sites in circCDYL luciferase reporter. **D.** Relative expression of miR-1275 in MCF-7 and MDA-MB-231 after circCDYL silencing or overexpression. **E.** Dual luciferase assay of HEK-293T cells co-transfected with miR-1275 mimic and luciferase reporter containing full length of linear CDYL. **G.** The quantification of mCherry dots and GFP dots in Fig 4F**. H.** Cell viability assay of MCF-7 or MDA-MB-231 cells after transfection with miR-1275 inhibitor or mimic**. I.** Autophagosomes in mCherry-GFP-LC3-labeled MDA-MB-231 after co-transfection with circCDYL over-expressing plasmid and miR-1275 mimic or co-transfection with circCDYL specific siRNA and miR-1275 inhibitor. Scale bar = 30μm. All data are shown as the mean ± S.E.M. **P* < 0.05, ***P* < 0.01, ****P* < 0.005.


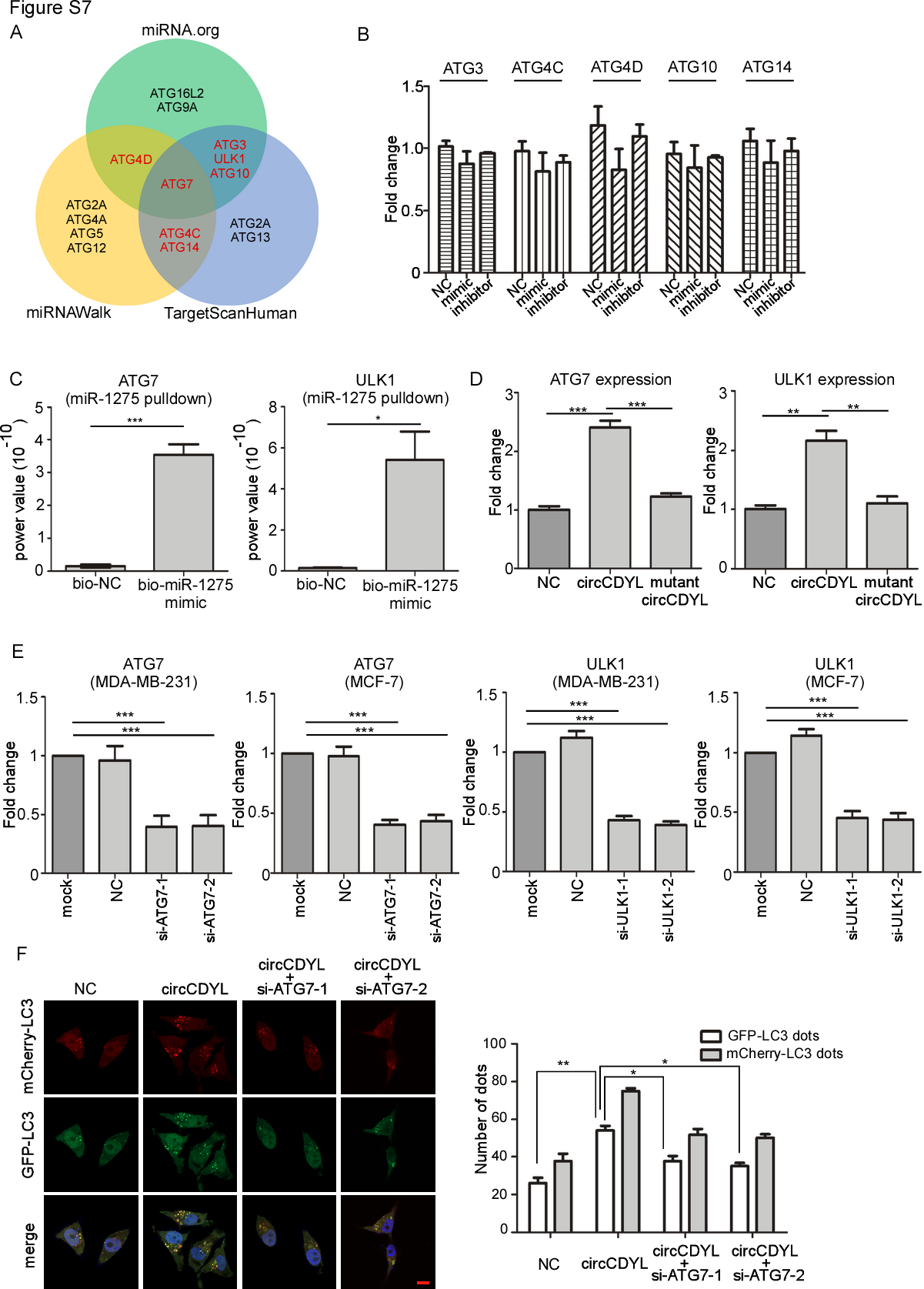
 **Fig S7. miR-1275 targets 3’ UTR of ATG7 mRNA. A.** Target Scan, miRNA.org and miRNAWalk online websites were employed to screen the potential miR-1275-targeted autophagy-associated genes. **B.** qRT-PCR analysis of ATG3, ATG4C, ATG4D, ATG10 and ATG14 expression in miR-1275 mimic- or inhibitor-transfected MDA-MB-231 cells. **C.** qRT-PCR analysis of ATG7 and ULK1 mRNA levels in RNA sample by miR-1275 miRNA pull-down. **D.** qRT-PCR analysis of ATG7 and ULK1 mRNA expression in circCDYL-wt (wide type) or circCDYL-mu (mutant) tranfected MDA-231 cells. circCDYL-mu contains two mutant miR-1275 binding sites. **E.** qRT-PCR analysis of mRNA level of ULK1 and ATG7 in ULK1- or ATG7-silenced MDA-MB-231 and MCF-7 cells. **F.** Autophagosomes in mCherry-GFP-LC3-labeld MDA-MB-231 after co-transfection with circCDYL over-expressing
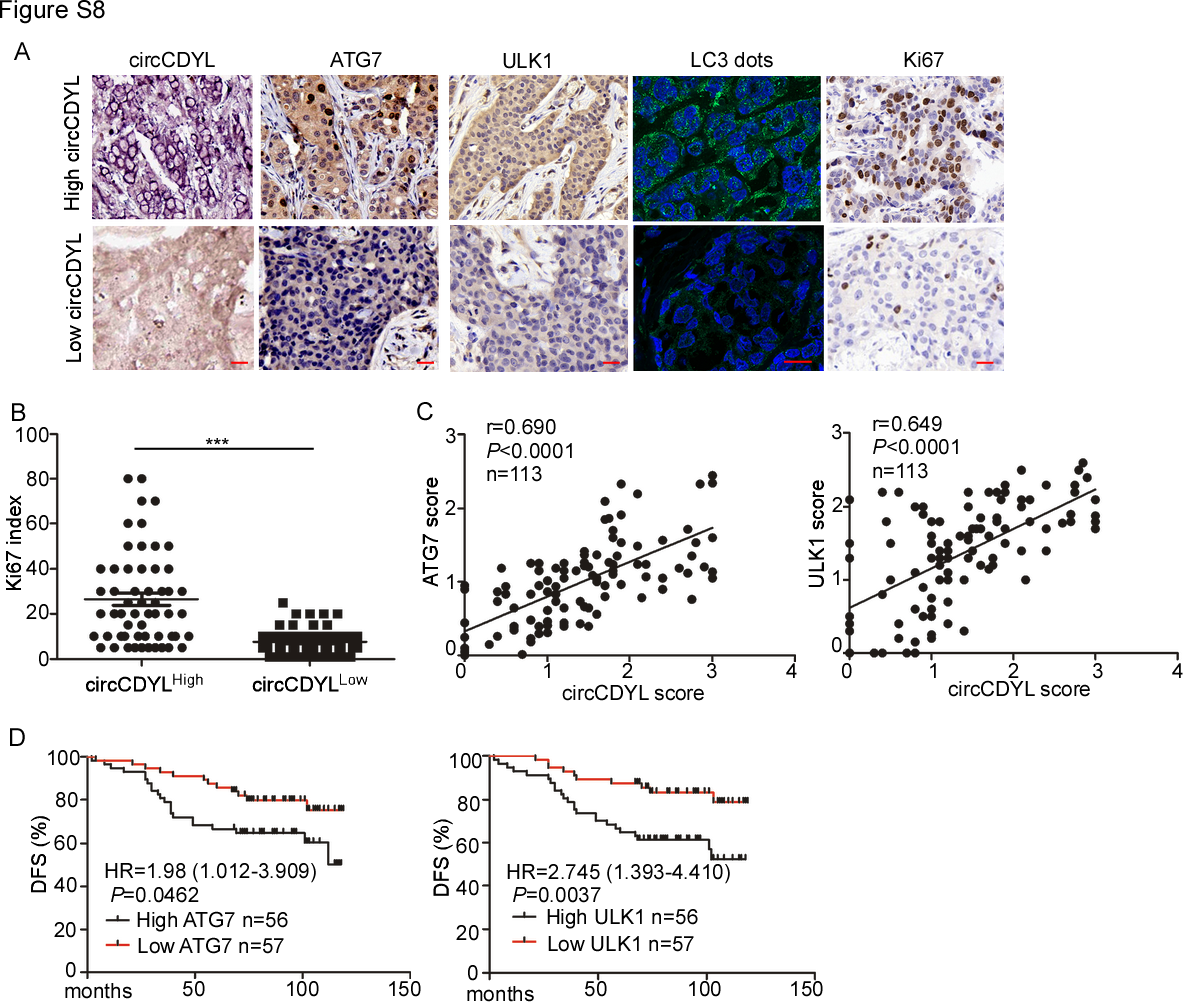
plasmids and ATG7 siRNA. Scale bar 30 μm.

**Fig S8. ATG7 and ULK1 protein expression in the SYSMH Cohort 1 with 113 breast cancer patients.** All experiments below were performed on the paraffin-embedded sections from Cohort 1(n=113). **A.** Detection of circCDYL by ATG7, ULK1 and Ki67 by IHC, LC3 dots by IF. Scale bar = 30μm. **B.** circCDYL expression was positively associated with Ki67 index. **C.** circCDYL expression was positively associated with ATG7 and ULK1 expression. **D.** Protein expression of ATG7 and ULK1 was associated with a poor disease-free survival (DFS) of BC patients.


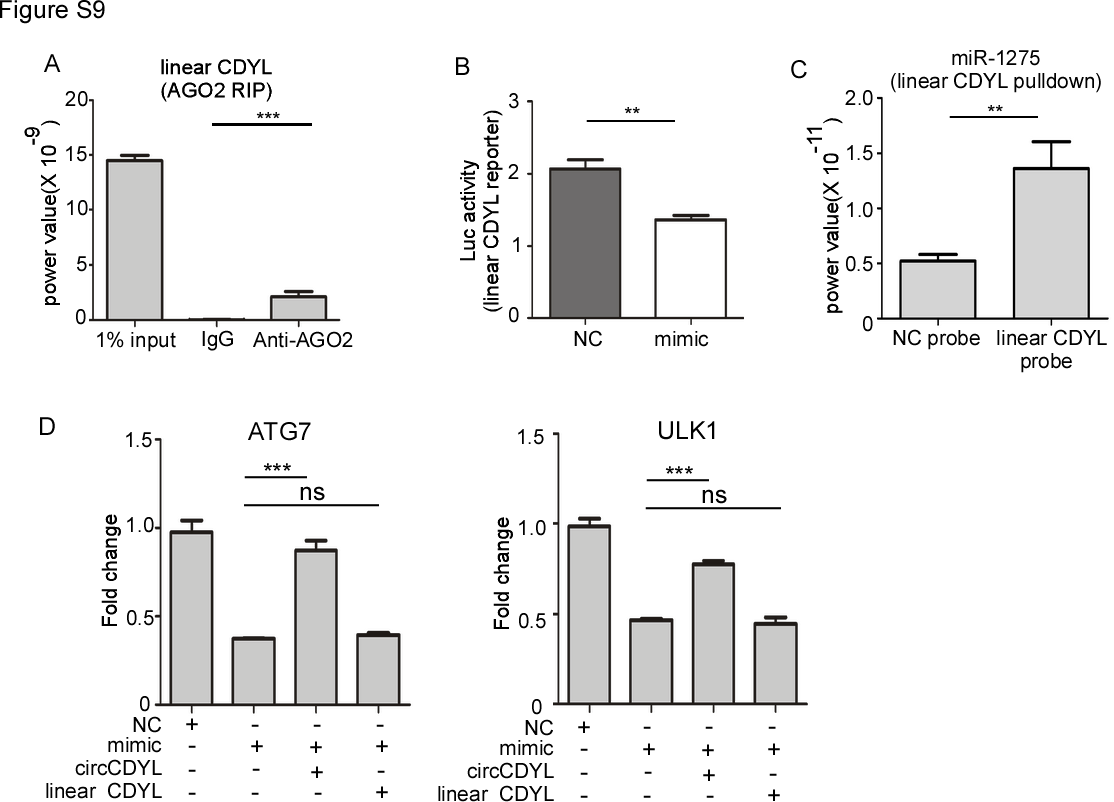
**Fig S9. The miR-1275 binding power of linear CDYL. A.** qRT-PCR analysis of linear CDYL in RNA sample after RIP assay by AGO2 antibody. **B.** Dual luciferase assay of HEK-293T cells co-transfected with miR-1275 mimic and luciferase reporter containing full length of linear CDYL. **C.** qRT-PCR analysis of miR-1275 in RNA sample by circCDYL pull-down. **D.** qRT-PCR analysis of ATG7 and ULK1 expression in MDA-MB-231, which were co-transfected with miR-1275 mimic and circCDYL or linear CDYL over-expressing plasmid.


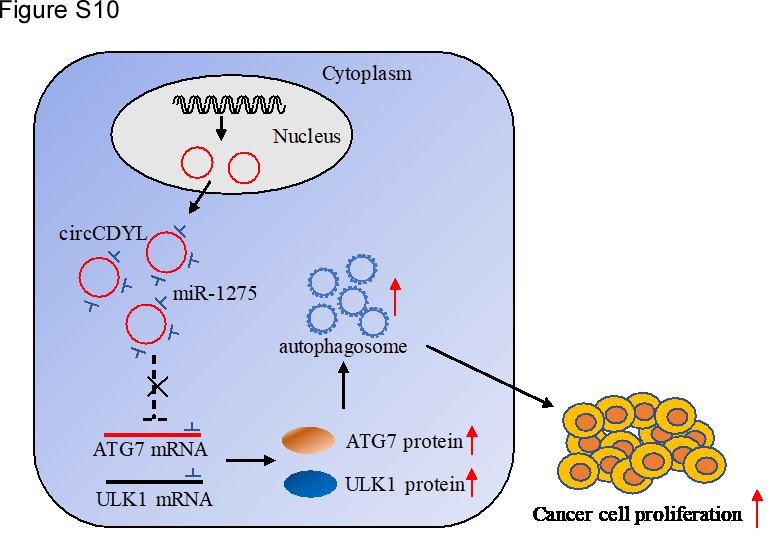
**Fig S10. Graphic abstract.** circCDYL is derived from the fourth exon of CDYL gene, and works as miR-1275 sponge in cytoplasm. circCDYL inhibits the activity of miR-1275 and promotes expression of ATG7 and ULK1, which is the target gene of miR-1275, thus to promote autophagosome formation and proliferation of BC cells.

| **Table S1. circRNAs deep sequencing in breast cancer tissue with different autophagic level** | | | | | | | | | | | | | | | |
| --- | --- | --- | --- | --- | --- | --- | --- | --- | --- | --- | --- | --- | --- | --- | --- |
| **circBase** | **gene_id** | **BC^*^ tissues with more LC3 dots (RPM^#^)** | | | | |  | **BC^*^ tissues with less LC3 dots (RPM^#^)** | | | | | ***P* value^&^** | **Expression** | **Fold Change** |
|  |  | **H1** | **H2** | **H3** | **H4** | **H5** |  | **L1** | **L2** | **L3** | **L4** | **L5** |  |  |  |
| hsa_circ_0008285  (circCDYL) | CDYL | 4333 | 4209 | 4076 | 3007 | 2912 |  | 1882 | 1871 | 1684 | 1599 | 1539 | 0.000233 | 2711.2 | 2.16 |
| hsa_circ_0008732 | BNC2 | 1192 | 721 | 491 | 1726 | 580 |  | 2558 | 2551 | 2517 | 1795 | 1730 | 0.002609 | 2230.116 | 0.42 |
| hsa_circ_0008838 | ZNF430 | 0 | 0 | 0 | 1703 | 1456 |  | 1614 | 2000 | 1718 | 1848 | 1151 | 0.037278 | 1666.218 | 0.38 |
| hsa_circ_0008359 | TMEM135 | 2418 | 555 | 1059 | 1294 | 2322 |  | 370 | 540 | 756 | 506 | 720 | 0.033238 | 1529.844 | 2.64 |
| hsa_circ_0040823 | BANP | 2264 | 2033 | 849 | 1036 | 1202 |  | 928 | 856 | 533 | 679 | 566 | 0.031106 | 1476.73 | 2.07 |
| hsa_circ_0024604 | ARHGEF12 | 0 | 0 | 695 | 690 | 659 |  | 1030 | 1243 | 1385 | 1066 | 1262 | 0.002315 | 1197.102 | 0.34 |
| hsa_circ_0001112 | DGKD | 1754 | 855 | 884 | 1571 | 873 |  | 440 | 457 | 761 | 564 | 696 | 0.019043 | 1187.462 | 2.03 |
| hsa_circ_0007059 | ZNF720 | 1542 | 631 | 1607 | 0 | 522 |  | 0 | 0 | 0 | 0 | 0 | 0.024286 | 860.248 | - |
| hsa_circ_0079480 | ISPD | 229 | 309 | 218 | 836 | 388 |  | 880 | 698 | 899 | 683 | 1083 | 0.010379 | 848.532 | 0.47 |
| hsa_circ_0006990 | VAPA | 539 | 1478 | 870 | 913 | 420 |  | 445 | 428 | 285 | 360 | 394 | 0.03854 | 843.89 | 2.21 |
| hsa_circ_0001386 | CTBP1 | 499 | 1173 | 912 | 291 | 438 |  | 300 | 276 | 281 | 345 | 185 | 0.049028 | 662.562 | 2.39 |
| hsa_circ_0000392 | YAF2 | 642 | 779 | 407 | 627 | 427 |  | 0 | 0 | 357 | 299 | 105 | 0.00329 | 576.422 | 3.79 |
| hsa_circ_0000230 | ZEB1 | 321 | 134 | 140 | 341 | 210 |  | 424 | 663 | 381 | 583 | 382 | 0.007457 | 486.304 | 0.47 |
| hsa_circ_0004365 | SEMA3C | 160 | 121 | 91 | 586 | 105 |  | 295 | 540 | 395 | 502 | 609 | 0.047365 | 468.234 | 0.45 |
| hsa_circ_0001079 | STK39 | 63 | 255 | 189 | 0 | 192 |  | 349 | 264 | 614 | 318 | 776 | 0.017859 | 464.038 | 0.3 |
| hsa_circ_0005589 | ARCN1 | 281 | 251 | 379 | 0 | 163 |  | 493 | 446 | 495 | 322 | 468 | 0.012159 | 444.776 | 0.48 |
| hsa_circ_0030883 | NAXD | 246 | 533 | 491 | 454 | 471 |  | 295 | 0 | 281 | 234 | 185 | 0.011054 | 439.09 | 2.21 |
| hsa_circ_0085173 | GRHL2 | 327 | 381 | 779 | 227 | 413 |  | 139 | 129 | 133 | 8 | 86 | 0.009923 | 425.21 | 4.29 |
| hsa_circ_0040827 | BANP | 201 | 479 | 379 | 350 | 388 |  | 161 | 117 | 95 | 180 | 0 | 0.001977 | 359.168 | 3.24 |
| hsa_circ_0072309 | LIFR | 57 | 63 | 0 | 186 | 130 |  | 70 | 428 | 285 | 422 | 542 | 0.016793 | 349.366 | 0.25 |
| hsa_circ_0042079 | GAS7 | 86 | 224 | 105 | 136 | 105 |  | 381 | 381 | 300 | 257 | 376 | 0.000382 | 338.832 | 0.39 |
| hsa_circ_0002465 | CD109 | 86 | 0 | 133 | 59 | 0 |  | 290 | 270 | 438 | 192 | 332 | 0.000829 | 304.248 | 0.18 |
| hsa_circ_0004228 | EVC2 | 201 | 112 | 42 | 91 | 58 |  | 354 | 364 | 314 | 318 | 160 | 0.002393 | 301.982 | 0.33 |
| hsa_circ_0005898 | MYOF | 149 | 125 | 112 | 141 | 167 |  | 338 | 323 | 247 | 215 | 363 | 0.000709 | 297.154 | 0.47 |
| hsa_circ_0000778 | EFCAB13 | 0 | 179 | 63 | 0 | 206 |  | 102 | 375 | 462 | 153 | 388 | 0.038235 | 295.992 | 0.3 |
| hsa_circ_0062649 | GRK3 | 0 | 139 | 84 | 127 | 174 |  | 182 | 287 | 305 | 257 | 400 | 0.004403 | 286.252 | 0.37 |
| hsa_circ_0006434 | SMG1 | 201 | 202 | 253 | 0 | 0 |  | 231 | 258 | 257 | 234 | 388 | 0.049415 | 273.462 | 0.48 |
| hsa_circ_0003057 | ANKH | 0 | 58 | 84 | 82 | 83 |  | 263 | 305 | 352 | 150 | 234 | 0.000756 | 260.662 | 0.24 |
| hsa_circ_0063019 | LARGE1 | 309 | 296 | 337 | 218 | 116 |  | 123 | 135 | 133 | 77 | 117 | 0.010277 | 255.138 | 2.18 |
| hsa_circ_0007095 | SETBP1 | 69 | 63 | 14 | 118 | 0 |  | 408 | 111 | 205 | 318 | 209 | 0.007324 | 250.234 | 0.21 |
| hsa_circ_0002563 | KIF2C | 395 | 322 | 147 | 213 | 134 |  | 97 | 129 | 38 | 127 | 0 | 0.019942 | 242.532 | 3.11 |
| hsa_circ_0008902 | PAN3 | 132 | 0 | 77 | 132 | 243 |  | 247 | 229 | 228 | 150 | 339 | 0.040464 | 238.384 | 0.49 |
| hsa_circ_0001383 | DLG1 | 338 | 202 | 140 | 236 | 254 |  | 145 | 35 | 152 | 100 | 0 | 0.01026 | 233.932 | 2.71 |
| hsa_circ_0005937 | DOCK4 | 132 | 85 | 77 | 91 | 112 |  | 263 | 188 | 343 | 265 | 105 | 0.01248 | 232.45 | 0.43 |
| hsa_circ_0008631 | UIMC1 | 132 | 0 | 0 | 0 | 152 |  | 220 | 188 | 200 | 253 | 302 | 0.002495 | 232.42 | 0.24 |
| hsa_circ_0007168 | HECTD2 | 40 | 81 | 239 | 50 | 112 |  | 236 | 270 | 247 | 215 | 142 | 0.023315 | 221.896 | 0.47 |
| hsa_circ_0001633 | SOBP | 172 | 130 | 42 | 100 | 33 |  | 182 | 358 | 171 | 192 | 191 | 0.022364 | 218.786 | 0.44 |
| hsa_circ_0008336 | BBS9 | 0 | 58 | 84 | 127 | 167 |  | 172 | 141 | 257 | 199 | 246 | 0.012427 | 202.984 | 0.43 |
| **BC, breast cancer*  *# RPM, junction reads per million reads*  *& P value detected by student’s t test* | | | | | | | | | | | | | | | |

| **Table S2. Patient characteristics stratified by expression of linear CDYL.** | | | | |
| --- | --- | --- | --- | --- |
| **expression of linear CDYL** | **Number of patients** | | **Total** | ***P* value^&^** |
|  | **Low** | **High** |  |  |
| **Age** |  |  |  | 0.326 |
| ＞35 | 49 | 53 | 102 |  |
| ≤ 35 | 7 | 4 | 11 |  |
| **Menopause** |  |  |  | 0.746 |
| Yes | 37 | 36 | 73 |  |
| No | 19 | 21 | 40 |  |
| **ER status** |  |  |  | 0.599 |
| Negative | 18 | 21 | 39 |  |
| Positive | 38 | 36 | 74 |  |
| **PR status** |  |  |  | 0.106 |
| Negative | 30 | 39 | 69 |  |
| Positive | 26 | 18 | 44 |  |
| **HER2 status** |  |  |  | 0.612 |
| Negative | 36 | 34 | 70 |  |
| Positive | 20 | 23 | 43 |  |
| **Ki 67 Level** |  |  |  | 0.098 |
| Low | 38 | 30 | 68 |  |
| High | 18 | 27 | 45 |  |
| **Molecular subtype** |  |  |  | 0.854 |
| Luminal | 38 | 36 | 74 |  |
| HER2+ | 8 | 10 | 18 |  |
| TNBC | 10 | 11 | 21 |  |
| **Tumor stage** |  |  |  | 0.243 |
| T1 | 23 | 15 | 38 |  |
| T2 | 23 | 28 | 51 |  |
| T3-4 | 10 | 14 | 24 |  |
| [**Lymphatic stage**](../../../../../AppData/Local/Youdao/Dict/Application/7.5.2.0/resultui/dict/) |  |  |  | 0.860 |
| N0 | 23 | 22 | 45 |  |
| N1 | 17 | 16 | 33 |  |
| N2-3 | 16 | 19 | 35 |  |
| *& P value detected by Chi-square test.* | | | | |

| **Table S3. miRNAs microarray after circRNA pull down** | | | | |  |  |  |  |  |  |
| --- | --- | --- | --- | --- | --- | --- | --- | --- | --- | --- |
|  | **Fold Change** |  | **Fore Group^*^** | |  | **ForeGround-BackGround^#^** | |  | **Normalized^&^** | |
| **Name** | **circCDYL vs NC** |  | **circCDYL** | **NC** |  | **circCDYL** | **NC** |  | **circCDYL** | **NC** |
| hsa-miR-1275 | 3.48 |  | 38334 | 10389 |  | 38281.5 | 10337.5 |  | 164.3 | 47.2 |
| hsa-miR-328-5p | 3.39 |  | 1244.5 | 380.5 |  | 1195.5 | 331 |  | 5.13 | 1.51 |
| hsa-miR-4657 | 3.18 |  | 418.5 | 159.5 |  | 368.5 | 109 |  | 1.58 | 0.5 |
| hsa-miR-149-5p | 3.01 |  | 72 | 58.5 |  | 24 | 7.5 |  | 0.1 | 0.03 |
| hsa-miR-328-5p | 2.92 |  | 10475 | 3406 |  | 10426 | 3357 |  | 44.75 | 15.33 |
| hsa-miR-3680-5p | 2.74 |  | 132 | 77.5 |  | 84.5 | 29 |  | 0.36 | 0.13 |
| hsa-miR-4728-5p | 2.65 |  | 3872.5 | 1404.5 |  | 3823 | 1354 |  | 16.41 | 6.18 |
| hsa-miR-149-3p | 2.61 |  | 9512 | 3457.5 |  | 9465 | 3409.5 |  | 40.62 | 15.57 |
| hsa-miR-4500 | 2.45 |  | 11233.5 | 4347 |  | 11182.5 | 4296.5 |  | 47.99 | 19.62 |
| hsa-miR-3127-5p | 2.21 |  | 470.5 | 230.5 |  | 419 | 178 |  | 1.8 | 0.81 |
| hsa-miR-1538 | 2.15 |  | 143.5 | 91 |  | 96 | 42 |  | 0.41 | 0.19 |
| hsa-miR-1228-5p | 2.12 |  | 345.5 | 180.5 |  | 298 | 132 |  | 1.28 | 0.6 |
| hsa-miR-4646-5p | 2.11 |  | 15663 | 7001.5 |  | 15614 | 6950 |  | 67.01 | 31.74 |
| hsa-miR-5000-3p | 2.08 |  | 572 | 286 |  | 525 | 237.5 |  | 2.25 | 1.08 |
| hsa-miR-5196-5p | 2.04 |  | 398.5 | 211 |  | 351.5 | 162 |  | 1.51 | 0.74 |
| hsa-miR-298 | 2.03 |  | 706 | 354.5 |  | 659 | 305 |  | 2.83 | 1.39 |
| ** The foreground intensity of each probe.*  *# The signal of the probe after background correction.*  *& The normalized ratio of the microRNA* | | | | | | | | | | |

| **Table S4. Sequence of siRNA or shRNA used in current stud** | | | |
| --- | --- | --- | --- |
| **Gene** | **No** | **Sense (5’ --3’)** | **Antisense (5’ --3’)** |
| circCDYL | si/sh-1 | GUUAACGGGAAAGGUUGAAdTdT | UUCAACCUUUCCCGUUAACdTdT |
|  | si/sh-2 | ACGGGAAAGGUUGAAAGGAUUdTdT | AAUCCUUUCAACCUUUCCCGTdTdT |
| linear CDYL | si-1 | GCCUACAGAUACAGAGAUAdTdT | UAUCUCUGUAUCUGUAGGCdTdT |
|  | si-2 | CGUUAUGUUUCCCAAGAUAdTdT | UAUCUUGGGAAACAUAACGdTdT |
| Linear + circular CDYL | si-1 | GGAAAGACCACGAAUCCAAdTdT | UUGGAUUCGUGGUCUUUCCdTdT |
|  | si-2 | GCACAUUGACCAGAACAAAdTdT | UUUGUUCUGGUCAAUGUGCdTdT |
| ATG7 | si-1 | CCAACACACUCGAGUCUUUdTdT | AAAGACUCGAGUGUGUUGGdTdT |
|  | si-2 | GCCUCUCUAUGAGUUUGAAdTdT | UUCAAACUCAUAGAGAGGCdTdT |
| ULK1 | si-1 | UCUCUAUAUGCAUAAAGUGdTdT | CACUUUAUGCAUAUAGAGAdTdT |
|  | si-2 | AUGUGUAUGUACAAACACCdTdT | GGUGUUUGUACAUACACAUdTdT |

| **Table S5. Primers used in current study** | | |
| --- | --- | --- |
| **Gene** | **Forward primer (5’ --3’)** | **Reverse primer (5’ --3’)** |
| circCDYL | ACCCACTAGTGCCTCAGGTG | TGTCGTCCTCGCTGTCATAG |
| Linear CDYL | ATTGCCTCTTTGCGATGT | TGTCAGCTTCCGTCCACT |
| ULK1 | AGCACGATTTGGAGGTCGC | GCCACGATGTTTTCATGTTTCA |
| ATG3 | GACCCCGGTCCTCAAGGAA | TGTAGCCCATTGCCATGTTGG |
| ATG4C | TAGAGGATCACGTAATTGCAGGA | GTTGTCAAAGCTGAGCCTTCTAT |
| ATG4D | GGAACAACGTCAAGTACGGTT | CTCGCCCTCGAAACGGTAG |
| ATG7 | CAGTTTGCCCCTTTTAGTAGTGC | CCAGCCGATACTCGTTCAGC |
| ATG10 | AGACCATCAAAGGACTGTTCTGA | GGGTAGATGCTCCTAGATGTGAC |
| ATG14 | GCGCCAAATGCGTTCAGAG | AGTCGGCTTAACCTTTCCTTCT |
| β-actin | TCATGAAGTGTGACGTGGACATC | CAGGAGGAGCAATGATCTTGATCT |
| GAPDH | AGGTGAAGGTCGGAGTCAAC | CGCTCCTGGAAGATGGTGAT |

| **Table S6. Probes used in current study** | | |
| --- | --- | --- |
| **Gene** | **FISH or ISH (5’ --3’)** | **Pull down (5’ --3’)** |
| MiR-1275 | GACAGCCTCTCCCCCAC | Patent of Synbio Company |
| circCDYL | CAATCCTTTCAACCTTTCCCGTTAAC | CAATCCTTTCAACCTTTCCCGTTAAC |
| Linear CDYL | GGAGTGGGTACTGATTCCAGACTCCAGATGCCTATT | GGAGTGGGTACTGATTCCAGACTCCAGATGCCTATT |
| NC | CCAGTGAATCCGTAATCATG | CCAGTGAATCCGTAATCATG |
| *NC, Negative control* | | |
